# Cold Acclimation in *Brachypodium* Is Accompanied by Changes in Above-Ground Bacterial and Fungal Communities

**DOI:** 10.3390/plants10122824

**Published:** 2021-12-20

**Authors:** Collin L. Juurakko, George C. diCenzo, Virginia K. Walker

**Affiliations:** 1 Department of Biology, Queen’s University, Kingston, ON K7L 3N6, Canada; george.dicenzo@queensu.ca (G.C.d.); walkervk@queensu.ca (V.K.W.); 2 Department of Biomedical and Molecular Sciences, School of Environmental Studies, Queen’s University, Kingston, ON K7L 3N6, Canada

**Keywords:** *Brachypodium distachyon*, cold acclimation, microbiome, amplicon and shotgun sequencing, metagenomics, *Pseudomonas*, *Streptomyces*

## Abstract

Shifts in microbiota undoubtedly support host plants faced with abiotic stress, including low temperatures. Cold-resistant perennials prepare for freeze stress during a period of cold acclimation that can be mimicked by transfer from growing conditions to a reduced photoperiod and a temperature of 4 °C for 2–6 days. After cold acclimation, the model cereal, *Brachypodium distachyon*, was characterized using metagenomics supplemented with amplicon sequencing (16S ribosomal RNA gene fragments and an internal transcribed spacer region). The bacterial and fungal rhizosphere remained largely unchanged from that of non-acclimated plants. However, leaf samples representing bacterial and fungal communities of the endo- and phyllospheres significantly changed. For example, a plant-beneficial bacterium, *Streptomyces* sp. M2, increased more than 200-fold in relative abundance in cold-acclimated leaves, and this increase correlated with a striking decrease in the abundance of *Pseudomonas syringae* (from 8% to zero). This change is of consequence to the host, since *P. syringae* is a ubiquitous ice-nucleating phytopathogen responsible for devastating frost events in crops. We posit that a responsive above-ground bacterial and fungal community interacts with *Brachypodium*’s low temperature and anti-pathogen signalling networks to help ensure survival in subsequent freeze events, underscoring the importance of inter-kingdom partnerships in the response to cold stress.

## 1. Introduction

As sessile organisms, plants are at the mercy of an array of abiotic stresses, and, as winter approaches in mid- to high-latitudes and altitudes, one such stress is low temperature. Plants employ various strategies that allow them to recognise and cope with the cold [1]. As autumn progresses, perennials undergo a period of cold acclimation, which in a few days of low temperature exposure allows them to physiologically prepare for freezing conditions. Such preparations include changed levels of hundreds of proteins, the accumulation of fatty acids, lipid remodelling for plasma membrane protection, increased production of cryoprotective metabolites, such as soluble sugars and amino acids, as well as chaperones and reactive oxygen scavengers [2]. This acclimation process also appears to coincide with changes in host-associated microbial communities. Such a turnover in microbiota could assist plants in preparing for sub-zero temperature conditions and their vulnerability to psychrophilic pathogens. Indeed, winter seasonality in the plant microbiome has been previously reported [3,4,5]. Although the impact of cold acclimation on the microbiomes of perennial grass has not been hitherto explored, the identification of their bacterial and fungal communities offers the promise of understanding how the battle against coming winter conditions can be won by partnerships.

The perennial grass and model cereal, *Brachypodium distachyon* (hereinafter, *Brachypodium*), is capable of cold acclimation, reaching peak freezing tolerance after two days at 4 °C, and is associated with changes in the abundance of multiple plasma membrane proteins at 2–6 days [6]. In turn, these proteins are involved in complex crosstalk networks that prime the *Brachypodium* defensive response to a variety of abiotic and pathogenic stresses. Studies of cold acclimation have, for the most part, ignored the host-associated microbiota [1,7,8]. Nevertheless, the plant microbiome is emerging as an important factor in stress responses, including symbiont-mediated tolerance [9,10].

The general beneficial effects of microbes on plant fitness under a variety of stressful conditions have recently come to be known as the “Defence Biome” [5,10,11,12,13,14,15,16,17]. Symbiont-mediated fitness benefits may be a collective result of microbial exudates and function, for example, by facilitating early stress sensing and more efficient nutrient uptake and transfer, as well as by the induction of plant stress genes [9,10]. Specifically, symbiont-mediated cold tolerance has been directly demonstrated with some plant species and plant growth promoting bacteria (PGPBs) [9]. For example, *Burkholderia phytofirmans*-inoculated grape vines expressed cold stress-responsive genes earlier than non-inoculated vines [18] and *Streptomyces neyagawaensis* J6-inoculated turfgrass showed enhanced cold tolerance over non-inoculated plants [19]. Microbes thus have a demonstrated role in plant protection. They excrete a variety of products to benefit host plants, including anti-pathogenic microbial compounds and osmolytes, including proline and trehalose, as well as scavengers of reactive oxygen species, such as superoxide dismutase, catalase, and peroxidases [9,10,20]. Taken together, plant-associated microbial communities undoubtedly help plants survive cold stress.

The identification of host-associated microbiota that enhance freezing tolerance may lead the way to the development of synthetic cocktails of species that could eventually be used to inoculate crops or seeds to enhance cold tolerance [21]. Here, shotgun sequencing and metagenomic analysis of the phyllosphere/endosphere and rhizosphere in cold-acclimated *Brachypodium* is an important first step towards this goal. Our experimental inoculation of a commercial growing mix with old pasture soil allowed for the exposure and subsequent identification of bacterial and fungal taxa that thrived after transfer of the growing plants to low temperatures and thus are prospective native partners in the cold acclimation process. In addition, we contribute to the general appreciation of the robustness of the plant abiotic stress response, which employs communities of diverse organisms for survival.

## 2. Materials and Methods

### 2.1. Soil Inoculation and Preparation

Commercial potting soil (Sun Gro Horticulture, Agawam, MA, USA) was autoclaved twice and sealed in a double layer of plastic autoclave bags before being inoculated with bulk field soil (5% *w*/*v*). Bulk field soil was sampled using a sterilized trowel from the active layer (3–7 cm depth) in autumn (29 October 2020) after 96 h of day and night temperatures of ~5 °C and ~0 °C, respectively. The sampled fallow field had been left unfertilized and unplowed for 26 years and without domestic grazing animals for 15 years (Figure S1). It was characterized by grasses, including orchard grass, brome, and timothy (*Dactylis*, *Bromus*, and *Phleum* species, respectively) on clay soils and was located north of Sydenham, Ontario, Canada (44°24′26″ N, 76°36′1″ W). Soils were thoroughly mixed for 15 min using a cement mixer that had been rinsed with 70% ethanol, with the inoculated soil then stored in a lidded container that had also been rinsed with 70% ethanol. The inoculated soil mixture was kept at room temperature until use.

### 2.2. Plant Material and Growth Conditions

Surface-sterilized *Brachypodium* seeds of an inbred line (ecotype: *Bd*21) (RIKEN, Wakō, Japan) were sown in the inoculated potting soil and grown in a temperature-controlled chamber (Conviron GEN2000, Queen’s University Phytotron, Kingston, ON, Canada) on a 20 h light (~100 µmol m^−2^ s^−1^; 22 °C) and 4 h dark (22 °C) light cycle. *Brachypodium* that had been grown under standard conditions for three weeks (Figure S2) were then cold acclimated by transferring the plants to a low temperature chamber (Coldmatic Refrigeration, Etobicoke, ON, Canada) (4 °C, 12 h light as indicated above; 12 h dark) for 6 days [6]. Plants maintained at standard conditions until time of use were considered the non-acclimated controls.

### 2.3. Microbiome Extraction and Preparation

Microbiome extractions were performed under sterile conditions. Above-ground extractions were from tissue excised from the tips of primary leaves. Phyllosphere microbes are found on the leaf surface and endosphere microbiota include communities that enter the plant through the leaves, as well as those that circulate within the xylem. Rather than separate these, we reasoned that both phyllosphere and endosphere communities would be driven by the changing environmental conditions, in addition to plant interactions. Accordingly, these leaf microbiota were extracted together using a DNeasy Plant Pro Kits (Qiagen, Hilden, Germany), following the manufacturer’s recommended directions, using 10 mg of leaf tissue per plant (10 plants per replicate for a total of 100 mg of tissue) and three replicates.

Extractions of the below-ground, tightly bound root soil of the rhizosphere (Figure S3) were performed as previously described [22] using a DNeasy PowerSoil Pro Kit (Qiagen, Hilden, Germany), following the manufacturer’s recommendations. Adhering root soil (25 mg per plant) was released from the roots following careful removal of the plants from the pots and gentle shaking. Extra care was taken to remove any root tissue, or non-soil material from samples, such as wood or perlite. Three replicates were performed, each using 10 individual plants. DNA purity and concentration was quantified using a Synergy H1 microplate reader with a Take3 Micro-Volume Plate (both BioTek Instruments Inc., Winooski, VT, USA).

### 2.4. Shotgun Metagenomics Library Preparation and Sequencing

Libraries were prepared using an Illumina DNA Prep (M) Tagmentation library preparation kit (Illumina Inc., San Diego, CA, USA), following the manufacturer’s user guide. Initial DNA concentration was evaluated using the Qubit dsDNA HS Assay Kit (Life Technologies, Carlsbad, CA, USA). Eukaryotic DNA was depleted in leaf tissue samples using an NEBNext Microbiome DNA Enrichment Kit (New England Biolabs, Ipswich, MA, USA), following the manufacturer’s user guide to decrease the probability of recovery of host genomic, chloroplast, and mitochondrial DNA sequences [23]. DNA (500 ng) was used for depletion of the eukaryotic DNA, as recommended by Molecular Research LP (MR DNA; Shallowater, TX, USA). The enriched microbial DNA was quantified using the Qubit dsDNA HS Assay Kit (Life Technologies, Carlsbad, CA, USA) (Table S1). Subsequently, 50 ng of DNA was used to prepare the libraries. The samples underwent simultaneous fragmentation and addition of adapter sequences, which were utilized during a limited-cycle polymerase chain reaction in which unique indices were added to the sample. Following library preparation, library concentration and mean library size were determined using the Qubit dsDNA HS Assay Kit (Life Technologies, Carlsbad, CA, USA) and the Agilent 2100 Bioanalyzer (Agilent Technologies, Santa Clara, CA, USA), respectively. Libraries were pooled in equimolar ratios (0.6 nM), and sequencing was performed on a NovaSeq 6000 platform (Illumina Inc., San Diego, CA, USA) to a depth of 10 million 2 × 150 bp reads.

### 2.5. Preprocessing and Quality Control

Analysis of sequencing data was performed following the Sunbeam pipeline (v2.1.0) [24] with 26 available cores (15.425 Gb of memory each) on Ubuntu (v18.04.05). Raw fastq files of paired-end reads were quality controlled to remove adapter sequences using Cutadapt (v3.4.0) [25] and Trimmomatic (v0.3.9) [26], following which read quality was assessed using FastQC (v0.11.9) [27]. Low-complexity sequences were masked using Komplexity (v0.3.6) [24] and contaminating plant host reads were removed by Sunbeam following mapping of reads to the *Brachypodium* genome (RefSeq assembly accession GCF_000005505.3) using BWA (v0.7.17) [28]. Following initial host read decontamination, individual reads were interrogated using the National Center for Biotechnology Information (NCBI) BLAST *(blastn;* available at https://blast.ncbi.nlm.nih.gov/Blast.cgi; accessed on 18 August 2021), revealing numerous hits to mitochondrial genomic sequences. Subsequently, several mitochondrial genomic sequences (detailed below) were subsequently downloaded and added to the host genome path for removal of contaminating mitochondrial sequences. This process was repeated until a subset of individual reads did not return any mitochondrial genomes with high coverage.

Most mitochondrial genomes used to filter contaminating sequences were retrieved from NCBI from the following species with GenBank IDs: *Saccharum officinarum* cv. Khon Kaen 3 (NC_031164.1), *Sorghum bicolor* (NC_008360.1), *Triticum aestivum* cv. Chinese Yumai (NC_036024.1), *Oryza sativa* (NC_011033.1), *Zea mays* (NC_007982.1), *Lolium perenne* (JX999996.1), *Oryza coarctata* (MG429050.1), *Sporobolus alterniflorus* (MT471321.1), *Aegilops speltoides* (AP013107.1), *Stipa capillata* (MZ161090.1, MZ161091.1, MZ161093.1, MZ161092.1), *Bambusa oldhamii* (EU365401.1), and a *Brachypodium* sequence (AC276583.1), suggesting a partial *Brachypodium* mitochondrial draft genome. In addition, the *Hordeum vulgare* mitochondria genome sequence was downloaded from Ensembl Plants (ID: IBSC_v2, chromosome Mt). Pre-processing and quality control data is summarized in Table S2.

### 2.6. Taxonomic Classification

Taxonomic assignment was performed on the quality-controlled and host-decontaminated reads using a Kraken2 (v2.1.2) [29] database containing RefSeq libraries [30] of archaea (628 sequences), bacteria (58,811 sequences), fungi (1579 sequences), and protozoa (11,151 sequences) for a total of 72,217 sequences and ~110 billion bp (as of 24 June 2021). A Bayesian re-estimation of abundance with the Kraken (Bracken) (v2.6) [31] database was subsequently built with the Kraken2 database using the default 35 k-mer length and 150 bp read lengths. Kraken2 was run as an integrated module of Sunbeam using the development branch. Bracken was run on the Kraken2 output files, and the Bracken outputs were combined using the combine_bracken_outputs.py function for downstream analysis. Barplots were produced using the thresholds indicated in the legends to group together low abundant taxa for visual presentation. For diversity analysis, the kraken-biom tool (v1.0.1) (https://github.com/smdabdoub/kraken-biom; accessed on 27 September 2021) was used to convert Bracken outputs at the species level into .biom files for use with the Phyloseq (v1.36.0) [32] and Vegan (v2.5.7) [33] R packages.

### 2.7. Core and Functional Microbiome

To further characterize the microbiomes, PAST (Paleontological Statistics, v4.08, available at https://www.nhm.uio.no/english/research/infrastructure/past/; accessed on 15 November 2021) [34] was used for similarity percentage (SIMPER) analyses using the Bray–Curtis similarity matrix to compare leaf and rhizosphere-associated microbiota and to facilitate the identification of a core microbiome [35,36,37]. Core microbiomes were calculated based on species and ASVs present in 100% of the tissue-specific samples with >5% relative abundance.

Paired-end quality-controlled and decontaminated reads outputted by Sunbeam were concatenated using the command “cat sample_R1.fq sample_R2.fq > merged_sample.fq” and inputted into HUMAnN (v3.0.0) [38] running MetaPhlan (v3.0) [38], Bowtie2 (v2.4.4) [39], DIAMOND (v2.0.11) [40], and SAMtools (v1.13) [41,42]. Sequences were processed using the default UniRef90 database and the following parameters for MetaPhlAn: --stat_q 0, --bt2_ps very-sensitive-local; the following parameters for HUMAaN 3: --nucleotide-subject-coverage-threshold 5.0, --translated-subject-coverage-threshold 5.0; and the following parameters for and Bowtie 2: -D 20 -R 3 -N 1 -L 20 -i S,1,0.50 --local.

Gene families were regrouped and renamed to the uniref90_Pfam database using the humann_regroup_table and humann_rename_table commands. Special features, including ungrouped genes and unintegrated pathways, were retained by skipping normalization in favour of downstream normalization using MaAsLin2 (v1.6.0) [43]. The final renamed gene family and unnormalized pathway abundance tables were joined using the humann_join_table command and split into the stratified and unstratified tables using the humann_split_table command, the latter of which was used for differential abundance testing. Standard HUMAnN3 MetaCyc assigned metabolic pathways were used for analysis and were assigned classes based on the respective associated MetaCyc pathway superclasses. All scripts can be found in Supplementary File S1.

### 2.8. Amplicon Sequencing

Aliquots of the DNA extractions used for shotgun sequencing were sent to MR DNA for amplification and barcoded amplicon sequencing of the 16S rRNA V4 region using primers 515F (5′-GTGYCAGCMGCCGCGGTAA-3′) [44] and 806R (5′-GGACTACNVGGGTWTCTAAT-3′) [45], and of the ITS region using primers ITS1F (5′-CTTGGTCATTTAGAGGAAGTAA-3′) and ITS2R (5′-GCTGCGTTCTTCATCGATGC-3′) [46]. Peptide nucleic acid clamps pP01 (5′-GGCTCAACCCTGGACAG-3′), as previously described [47], were used to reduce amplification of *Brachypodium-*contaminating sequences during the amplification of the 16S rRNA V4 regions. Blank kit controls for both Plant Pro and PowerSoil Pro kits were performed in triplicate and subjected to the same amplification and sequencing as the corresponding samples. Sequencing was performed on a MiSeq platform (Illumina Inc., San Diego, CA, USA) for ITS and NovaSeq 6000 platform (Illumina Inc., San Diego, CA, USA) for 16S.

### 2.9. Amplicon Sequence Processing

Sequences were processed using QIIME2 (v2021.4) [48]. Raw .fastq files were demultiplexed and non-biological sequences were removed, including primers, adapters, spacers, and linkers, using FASTqProcessor (v20.11.19). Sequences were trimmed and denoised to remove any chimeras and singletons using DADA2 (v1.18) [49] before being grouped into amplicon single variants (ASVs). ASVs were used for taxonomic classification with SILVA (v138) for 16S rRNA sequences and UNITE (v8) for ITS sequences [50,51,52,53,54,55]. In the leaf samples, any taxa classified as eukaryota, chloroplast, mitochondria, archaea, or unclassified were filtered out of the 16S rRNA feature tables. Shannon’s diversity index was used as a measure for alpha diversity and Bray–Curtis dissimilarity distance was used as a measure for community dissimilarity. Principal coordinate analysis (PCoA) was performed using Bray–Curtis dissimilarity matrices and plots made in R using ggplot2. Differential abundance between cold-acclimated and non-acclimated samples and between blank kit controls and samples was also assessed at the genus taxonomic levels using ANCOM-BC in R (v1.2.2) [56]. All commands and codes used can be found in Supplementary File S1.

### 2.10. Statistical Analysis

All statistical analyses were performed in RStudio (v1.3.1073) running R (v4.1.1) and all scripts used are available in Supplementary File S1. All plots, when necessary, were cleaned up using Inkscape (v0.92.2). Alpha and beta diversity analysis was performed using the Vegan and Phyloseq packages and PCoA plots were performed using ggplot2 (v3.3.5). To find differentially abundant taxa between the two temperature conditions, ANCOM-BC was run on Bracken outputs with default parameters for shotgun data and feature tables for amplicon data. Output coefficients representing the natural log fold-change model were converted to log2 fold changes. ANCOM-BC outputs were parsed to remove any low abundant taxa from differential abundance results.

## 3. Results

### 3.1. Pre-Processing, Shotgun Sequencing, and Kit Controls

Initial DNA samples representing the cold-acclimated (CA) leaf and rhizosphere were sent for shotgun sequencing without eukaryotic depletion, revealing high host contamination in the leaves (not shown). Subsequent replicate samples undergoing eukaryotic depletion proved successful as the classification of processed reads showed a full order of magnitude better recovery of microbial sequences. DNA and library concentrations and average size, quality control, host read decontamination, and Kraken2 classification results are summarized in the Supplementary Materials (Figure S4, Tables S1 and S2). Although shotgun DNA library construction was attempted on the blank kit controls, a lack of sufficient DNA resulted in no results for this sequencing method. However, the same control samples were subject to amplicon marker gene sequencing. Following QIIME2 processing, it was determined through diversity analysis and PCoA using Bray–Curtis dissimilarities that the microbial compositions associated with the kits were significantly different than the *Brachypodium* leaf (*p* < 0.001 16S, *p* < 0.05 ITS, pairwise PERMANOVA) and rhizosphere microbiomes (*p* < 0.001 16S, *p* < 0.05 ITS, pairwise PERMANOVA) (Figure S5).

### 3.2. Compatible Results with Shotgun and Amplicon Sequencing

The correlation between taxa identified in both the shotgun data and the amplicon data was assessed at the genus level in order to compare the two methods. In the CA rhizosphere, the genera identified by shotgun metagenomic and 16S rRNA amplicon sequences, as well as shotgun metagenomics and ITS amplicon sequencing, were well correlated (R^2^ = 0.93 and R^2^ = 0.88, respectively) (Figure S6). The non-acclimated (NA) rhizosphere shotgun and 16S rRNA, and the shotgun and ITS amplicon results (R^2^ = 0.91 and R^2^ = 0.45, respectively) also correlated, but less well. It is notable that for the leaf microbiome, bacterial taxa in the CA shotgun and 16S rRNA samples, as well as for the NA leaf samples, showed mixed correlations (R^2^ = 0.31 and R^2^ = 0.75, respectively). Insufficient fungal reads in the leaves following Bracken re-estimation resulted in no correlation between the shotgun and ITS reads in the leaves.

### 3.3. Cold Acclimation and the Rhizosphere Microbiome

**Figure 1:**
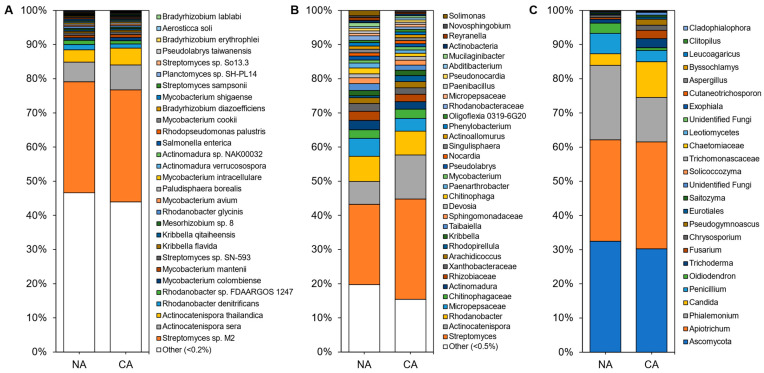
Average relative abundance of the taxonomies of the non-acclimated and cold-acclimated *Brachypodium distachyon* rhizosphere microbiomes: (**A**) species identified from shotgun sequencing and metagenomics classified using a custom Kraken2 database, (**B**) distinct amplicon sequence variants assigned down to the genus or lowest possible level by QIIME2 using the SILVA database for 16S rRNA sequences amplified using the V4 region of prokaryotes, and (**C**) distinct amplicon sequence variants assigned down to the genus or lowest possible level by QIIME2 using the UNITE database for ITS regions of eukaryotes.

In total, 4646 microbial species were identified in the rhizosphere shotgun data with 45 ± 3% of reads remaining unclassified. The majority of identified reads, 99.70 ± 0.06%, represented bacterial microbes with 0.15 ± 0.03% and 0.13 ± 0.02% representing fungi and archaea, respectively. Alpha diversity, assessed using Shannon’s diversity index, across all rhizosphere samples was 4.98 ± 0.21 and was not significantly different between conditions with 5.07 ± 0.29 in the CA and 4.91 ± 0.94 in the NA samples. The rhizosphere was dominated by *Streptomyces* sp. M2, a PGPB, accounting for approximately one-third of the taxa in all samples. Rounding out the top abundant species across the rhizosphere samples were taxa present at 1–10% abundance, which included *Actinocatenispora sera*, *Actinocatenispora thailandica*, *Rhodanobacter denitrificans*, and *Rhodanobacter* sp. *FDA-ARGOS* 1247 (Figure 1A; Table S3). Nearly half of all species in the rhizosphere shotgun data were below a cut-off value (0.2%) for low relative abundance leaving a balance of 53% and 56% of species found in NA and CA samples, respectively.

The amplicon analysis identified 651 distinct ASVs at the genus level. Alpha diversity appeared similar in the NA and CA samples (6.79 ± 0.25 and 6.40 ± 0.16, respectively) and differences were not significant. Both conditions were dominated by the genera *Streptomyces*, *Actinocatenispora*, and *Rhodanobacter (*Figure 1B; Table S3*)*. After CA, low abundant taxa (<1% relative abundance) remained equal at 29%. Again, a similar number of ASVs were considered at low abundance under NA and CA conditions (20% and 15%, respectively). ITS analysis showed 25 distinct ASVs at the genus level (Figure 1C). *Ascomycota* and *Apiotrichum* each represented a third of the ASVs in the rhizosphere irrespective of conditions (Figure 1C; Table S3). Alpha diversity was significantly different (*p* < 0.05, two-tailed *t*-test) at 3.43 ± 0.06 in the CA and 3.05 ± 0.17 in the NA.

**Figure 2:**
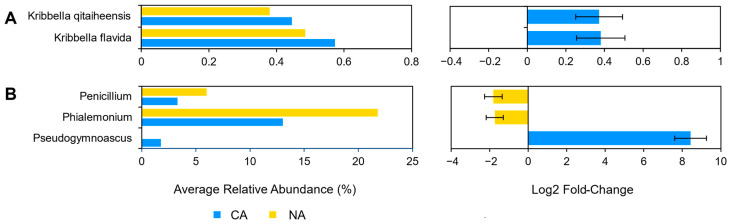
Differentially abundant taxa between the non-acclimated and cold-acclimated *Brachypodium distachyon* rhizosphere microbiomes as determined by ANCOM-BC and showing their average relative abundance in both conditions and log2 fold changes with error bars representing standard error: (**A**) species identified by Kraken2 from shotgun sequencing data that are differentially abundant and above an average relative abundance threshold of 0.2%, and (**B**) ITS amplicon sequence variants that are differentially abundant. Only statistically significant changes are shown, as determined by ANCOM-BC.

Although there were few changes in the rhizosphere community following 6 days at 4 °C, differential abundance testing using ANCOM with bias control and parsed for taxa above the assigned low relative abundance thresholds (Figure 1) identified two modestly differentially abundant species (out of 143; 1.4%) in the shotgun data. *Kribbella qitaiheensis* (log2 fold change: 0.37) and *Kribbella flavida* (log2 fold change: 0.38) increased in relative abundance after CA (Figure 2A). In addition, the relative abundance of three fungal genera (out of 25; 12%) changed following CA, including a decrease in *Penicillium* (log2 fold change: −1.8) and *Phialemonium* (log2 fold change: −1.7) and a more substantial relative increase in *Pseudogymnoascus* (log2 fold change: 8.43) (Figure 2B).

**Figure 3:**
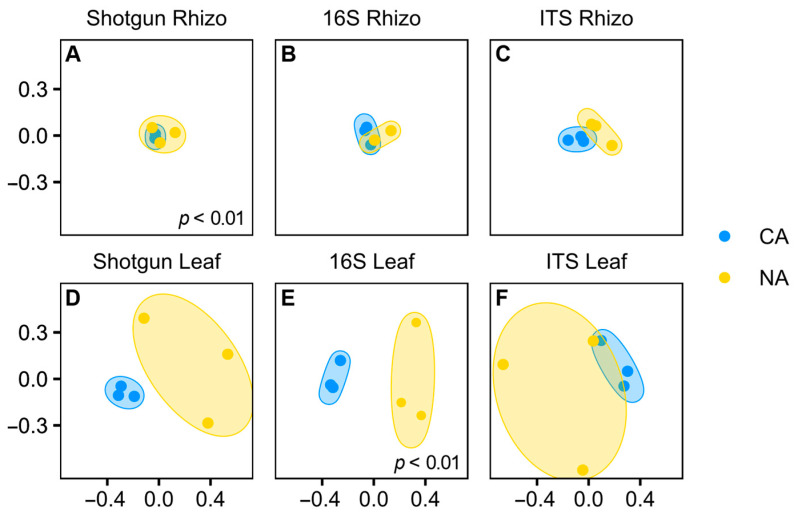
Principal coordinate analysis comparing non-acclimated and cold-acclimated conditions in each sample type for each sequencing method, for the following samples: (**A**) shotgun sequencing in the rhizosphere, (**B**) 16S rRNA sequencing of the V4 region in the rhizosphere, (**C**) ITS sequencing of the rhizosphere samples, (**D**) shotgun sequencing of the leaf samples, (**E**) 16S rRNA sequencing of the V4 region in the leaf samples, and (**F**) ITS sequencing of the leaf samples. Pairwise PERMANOVAs were conducted between conditions with significance as noted.

Although shifts in the rhizosphere community appeared modest, the Bray–Curtis dissimilarity analysis showed that the shotgun rhizosphere communities were significantly different under the two temperature regimes (*p* < 0.01, pairwise PERMANOVA) (Figure 3A). In contrast, there were no differences in Bray–Curtis dissimilarity for the amplicon analysis, either for 16S (Figure 3B) or ITS data (Figure 3C). Taking all the results together, it appears that overall, the CA regime resulted in only a very minor shift in the rhizosphere microbial community. We speculate that a longer period of low temperature with concomitant changes in root exudates would be required for a more dramatic change in the root-associated microbiota.

### 3.4. Cold Acclimation and the Leaf Microbiome

**Figure 4:**
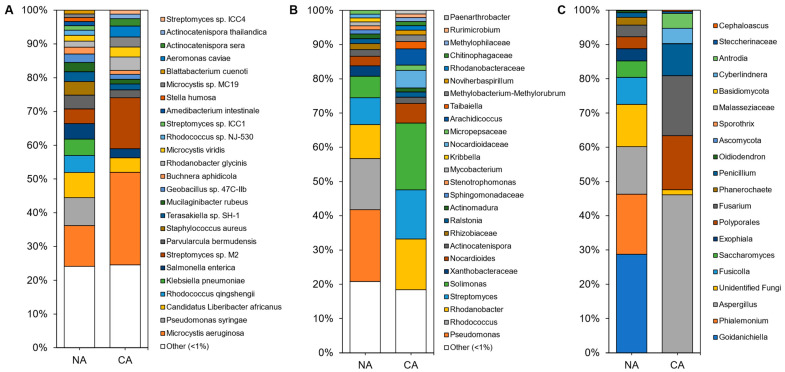
Average relative abundance of the taxonomies of the non-acclimated and cold-acclimated *Brachypodium distachyon* leaf microbiomes representing the endosphere and phyllosphere: (**A**) species identified from shotgun sequencing and metagenomics classified using a custom Kraken2 database, (**B**) distinct amplicon sequence variants assigned down to the genus or lowest possible level by QIIME2 using the SILVA database for 16S rRNA sequences, amplified using the V4 region of prokaryotes, and (**C**) distinct amplicon sequence variants assigned down to the genus or lowest possible level by QIIME2 using the UNITE database for ITS regions of eukaryotes.

Although shotgun sequencing of the leaf, representing the endosphere and phyllosphere microbiomes, identified 143 microbial species with the most abundant taxa shown (Figure 4A; Table S4), an average of 92 ± 4% of the reads remained unclassified, with a portion of these likely attributable to as yet unsequenced host mitochondrial sequences (Figure S4C). Bacteria accounted for ~100% of the microbiota except in a couple of samples from which a few fungal sequences were recovered. Overall, alpha diversity was significantly lower (*p* < 5 × 10^−6^, two-tailed *t*-test) in leaf samples (3.18 ± 0.36) compared to rhizosphere samples (4.99 ± 0.21).

Leaf alpha diversity did not significantly change after CA treatment (mean Shannon indices at 3.30 ± 0.29 in NA samples and 3.06 ± 0.47 in CA samples). However, the taxa profile changed with the cyanobacteria *Microcystis aeruginosa*, decreasing from ~27% to ~13% relative abundance after CA. *Streptomyces* sp. M2 showed the opposite profile, increasing from ~4% to ~15% average relative abundance after transfer to 4 °C. NA leaves were dominated by the plant pathogens *Pseudomonas syringae* and ‘*Candidatus* Liberibacter africanus’, as well as the plant beneficial *Rhodococcus qingshengii,* whose levels substantially decreased in the CA conditions. Lower abundant reads (<1%) made up about a quarter of the taxa, similar to the CA samples.

Amplicon sequencing of the 16S rRNA from the leaves identified 188 distinct ASVs at the genus level (with the most abundant shown in Figure 4B and Table S4). Again, alpha diversity was not significantly different between conditions (5.04 ± 0.25 and 4.60 ± 0.70 in the CA and NA samples, respectively). Taxa present under both conditions included the genera *Solimonas*, *Rhodanobacter*, and *Streptomyces*. *Pseudomonas* and *Rhodococcus* were abundant (21% and 15% average relative abundance, respectively) in NA conditions, but decreased in relative abundance after transfer of the plants to 4 °C with log2 fold changes of −4.18 and −5.41, respectively. The cereal growth-promoting genus *Nocardioides* and an unidentified genus from the same family, *Nocardioidaceae*, both increased in abundance to represent 11% of the taxa in CA plants. ASVs at low relative abundance (<1%) made up a similar 18% and 21% of CA and NA 16S samples, respectively. ITS analysis resulted in 20 distinct ASVs at the genus level (Figure 4C).

**Figure 5:**
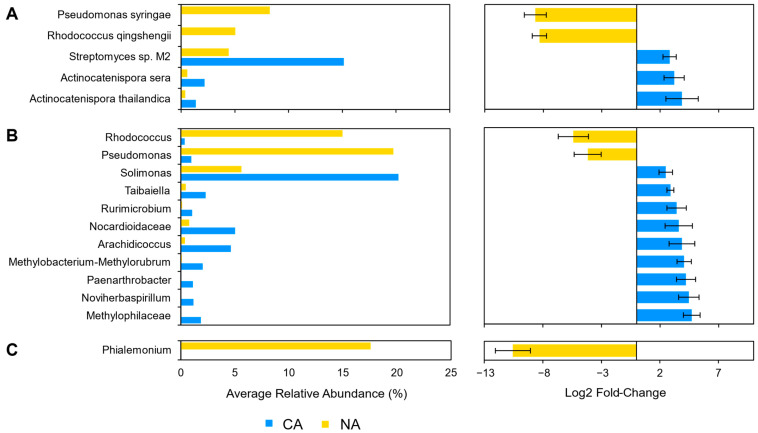
Differentially abundant taxa between the non-acclimated and cold-acclimated *Brachypodium distachyon* leaf microbiomes representing the endosphere and phyllosphere as determined by ANCOM-BC and showing their average relative abundance in both conditions and log2 fold changes with error bars representing standard error: (**A**) species identified by Kraken2 from shotgun sequencing data that are differentially abundant and above an average relative abundance threshold of 1%, (**B**) distinct 16S rRNA amplicon sequence variants assigned by QIIME2 and the SILVA database to the genus level that are differentially abundant, and (**C**) distinct ITS amplicon sequence variants assigned by QIIME2 and the UNITE database to the genus level that are differentially abundant. Only statistically significant changes are shown as determined by ANCOM-BC.

After shotgun sequence analysis, 3.5% (5/143) of the taxa were identified as differentially abundant between the NA and CA conditions (Figure 5A). After transfer to 4 °C, reads attributed to *P. syringae* (log2 fold change: −8.68) and *R. qingshengii* (log2 fold change: −8.33) decreased so that there was a change in the estimated average relative abundance of *P. syringae* and *R. qingshengii* from 8.2% and 5.0% to 0%, respectively. At the same time there was a corresponding increase in the relative abundance of *Streptomyces* sp. M2 (log2 fold change: 2.81), *A. sera* (log2 fold change: 3.20), and *A. thailandica* (log2 fold change: 3.87). In 16S CA samples, nine other taxa increased, including the genus *Solimonas*, which increased in relative abundance but was below the low abundance threshold. In total, 5.9% (11/188) of the identified sequences above the threshold were found to be differentially abundant. For the ITS analysis, the genus *Phialemonium* represented 5% (1/20) of the ASVs and decreased in relative abundance (log2 fold change: −10.6) (Figure 5C).

Despite the apparent community differences, Bray–Curtis dissimilarity analysis suggested that the microbial communities identified with the shotgun sequencing approach were not significantly different, undoubtedly due to the low number of sequences (Figure 3D), similar to the leaf ITS communities. Supporting that conclusion, 16S rRNA communities were shown to be significantly different between conditions (*p* < 0.01, pairwise PERMANOVA) with the analysis supported by high ASV numbers (Figure 3E).

### 3.5. Dissimilarity Comparisons and Core Microbiome

The root and leaf-associated microbiomes were further independently characterized with SIMPER to identify taxa that contributed the most dissimilarity between NA and CA regimes (Table 1). For microbiota isolated from the rhizosphere, the taxa contributing to the top ~25% of dissimilarity were *Streptomyces* sp. M2, *A. sera*, and *A. thailandica* for the shotgun data, the genera *Actinocatenispora* and *Streptomyces* for the 16S data, and the genera *Phialemonium* and *Apiotrichum* for the ITS data. For leaf samples, taxa contributing to the top ~25% dissimilarity were *M. aeruginosa* and *Streptomyces* sp. M2 for the shotgun data, the genera *Pseudomonas* and *Rhodococcus* for the 16S data, and the genera *Aspergillus* and *Goidanichiella* for the ITS data.

Highly conserved taxa that are present in most samples, typically ~70%, can be considered part of the “core” microbiome that orchestrates the interactions between the host and the microbiota [57]. As described in the methods, we employed strict criteria that the taxa must appear in all of the samples for each condition (Table 2). In the rhizosphere, the core microbiota identified in the shotgun analysis included *Streptomyces* sp. M2 and *Actinocatenispora sera.* Core taxa in the leaves included *Streptomyces* sp. M2 and ‘*Candidatus* Liberibacter africanus’, both of which persisted across the two different conditions and all samples. The larger number of taxa associated with the rhizosphere ASVs were consistent with the microbes identified by shotgun analysis and indicated bacterial (*Streptomyces*, *Actinocatenispora*, and *Rhodanobacter*) as well as fungal taxa (*Ascomycota*, *Apiotrichum*, *Phialemonium*, and *Candida*) as contributors to the core microbiome. Leaf ASVs revealed that bacteria (*Streptomyces*, *Rhodanobacter*, and *Solimonas*), as well as a single unidentified fungal sequence, comprised the core.

## 4. Discussion

The plant-microbiome partnership is responsive to stress, with the details of the signalling between the kingdoms of Eubacteria, Fungi, and Planta only beginning to be investigated [9,10,58,59]. Sub-zero temperatures are a particular challenge, resulting in cellular dehydration, membrane rupture, and increased vulnerability to psychrophilic pathogens and death, but some perennials respond to earlier non-freezing temperatures, and/or shortened day lengths to initiate a signalling response. This CA stress triggers changes in plant metabolism, resulting in cold-hardening and survival during subsequent freeze events and is accompanied by significant changes in the leaf microbiome community profile, but with less substantial community shifts in the rhizosphere (Figure 1 and Figure 3).

### 4.1. Little Change in Rhizosphere Communities after Cold Acclimation

The different sequencing methodologies employed, either amplicon or shotgun analyses, generally yielded compatible results. As indicated, there were few changes in the rhizosphere community after the shift to low temperatures, as shown by the overlapping PCoA groupings with rare exceptions, and for the most part these did not make up a large proportion of the taxa. The rhizosphere communities from both NA and CA plants contained taxa previously reported in bound soils associated with *Brachypodium* and similar to those found in wheat [22]. Some species of the order *Burkholderiales* have been isolated from ryegrass rhizospheres and are associated with nutrient acquisition such that there is interest in their potential as beneficial probiotics for crop enhancement [60]. Ascomycota is dominant in grassland soils, which can be low in organic matter and nutrients, playing key roles in cyanobacteria-dominated soils as well as having important roles in cycling carbon and nitrogen in addition to nutrient transport [61]. The fact that these taxa are shared in wheat and *Brachypodium* underscores the co-evolution of the plant–host relationship, since microbiota in the dicot, *Arabidopsis*, is distinct [22]. As noted, neither the *Brachypodium* bacterial nor fungal communities changed significantly after the plants were moved to 4 °C, suggesting that there was insufficient time for the soil to reach that temperature. Indeed, investigations of cold-responsive rhizosphere microbiota in maize used 5 weeks exposure to “chilling” conditions compared to our 6-day treatment [17]. In addition, it is notable that the myriad of CA-dictated changes made in the above-ground portion of *Brachypodium* are not apparently signalled to the rhizosphere during the treatment regimen.

### 4.2. Shifts in Leaf Communities Accompany Cold Acclimation

Compared to the rhizosphere, which is relatively protected from rapid abiotic and biotic stresses, leaves are exposed to daily temperature fluctuations, visible and ultraviolet light, herbivore and mechanical damage, and arguably more pathogens. Within two days of the shift to CA conditions, the *Brachypodium* leaf membrane is protected from freeze-induced electrolyte leakage, contains elevated levels of soluble sugars, and shows changes in the abundance profiles of hundreds of proteins [6]. The leaf community response was also rapid, as revealed by numerous abundance changes in the bacterial and fungal microbiota, as well as in the proportion of individual core taxa, as supported by the distinct groupings shown in PCoAs (Figure 3 and Figure 5; Table 2). Similarly, cold-associated shifts occurred in leaves from European grasslands over winter while the rhizosphere was relatively unchanged [4]. As in the rhizosphere data, results from the two sequencing methods were generally consistent. However, a notable exception was for sequences corresponding to the toxic cyanobacteria *Microcystis aeruginosa*, which were abundant in NA and increased after CA, but only when using the shotgun methodology. It is possible that these sequences were misclassified as chloroplast DNA and were mistakenly filtered from the amplicon data. We speculate that the increase in relative abundance of cyanobacteria after CA is likely due to the reduction in evaporation on the leaf surfaces at low temperatures, consistent with their preference for aquatic habitats, and their known colonization of the phyllosphere [62].

For other taxa, there was clear evidence of a change in relative abundance after CA that was generally consistent irrespective of the sequencing methodology. This included three prominent *Actinobacteria* species that increased in relative read numbers, including the grassland-associated *Actinocatenispora thailandica* and *Actinocatenispora sera*, as well as the mycelium-producing *Streptomyces* sp. M2, a known PGPB [63]. Although present in the rhizosphere samples under both conditions, *Streptomyces* sp. M2 increased 216-fold in relative abundance following CA in leaves. Presumably, it promotes plant growth with its extensive repertoire of antibiotics, plant growth hormones, siderophores, and insecticides [63,64,65]. Strikingly, this *Streptomyces* strain can inhibit the plant pathogen *P. syringae*, perhaps due to siderophores that chelate iron required by *Pseudomonas* [63]. Such inhibition could explain the disappearance of *P. syringae* after CA treatment, representing a log2 fold change of −8.7.

Other bacteria also showed inverse abundance profiles depending upon the condition, as described in the Results section. Fungal ascomycete taxa similarly exchanged their relative abundance, with a decrease in the genus *Goidanichiella* and an increase in the genus *Aspergillus* detected after CA. These changes may be related to the temperature regime since *Goidanichiella* was reported to dominate summer-collected wheat leaves whereas cold-tolerant *Aspergillus* are of interest as growth promoters likely due to their ability to solubilize phosphates [66,67].

### 4.3. Leaf Cold Acclimation Associated with Low Temperature and Pathogen Responses

After transfer to 4 °C, the leaf microbiome was impacted by the temperature shift and also showed changes in the relative abundance of potential pathogens. These observations reflect the results of network analysis of hundreds of plasma membrane proteome changes after CA that showed crosstalk between pathways for low temperature stress and disease and defence [6]. *Brachypodium* responds to CA by diverting resources away from growth and to the stress response. It appears then that the host–microbiome works together in a joint effort to prepare for the worsening conditions associated with winter.

One of the most obvious examples of the connection between low temperature and disease is found in the ice nucleation-active plant pathogen *P. syringae*, which can facilitate the formation of ice at temperatures just below 0 °C, presumably to lyse plant cells and thus access nutrients [68]. In NA leaves, *P. syringae* was a large contributor to the bacterial taxa (8% of the shotgun reads). However, as the temperature drops, such a large proportion of *P. syringae* in the leaf microbiota would surely present a grave risk to the host plant. Remarkably, after CA there was no evidence of this bacteria. This disappearance is undoubtedly fostered by *Brachypodium*’s defence pathways that lead to the production of multiple proteins, including antifreeze proteins, that target the ice nucleator, but we propose that the microbiome also supports this protective strategy.

Coincident with the collapse of the *P. syringae* population, there was a 216-fold increase in the relative abundance of *Streptomyces* sp. M2 (0.1% to 15.1%). It is important to note that this increase after CA cannot be explained by sensitivity to the NA growth conditions since it is routinely cultured at 30 °C [69]. Thus, the change in its abundance is independent of the temperature shift and may be fostered by *Brachypodium*. As mentioned, this PGPB secretes antibiotics and siderophores and is known to inhibit *P. syringae* [63]. *Rhodococcus* also decreased 40-fold in relative abundance, but to date there is no information on its interaction with *Streptomyces* or other plant beneficials. Nevertheless, as well as directly targeting *P. syringae,* it is likely that *Streptomyces* alerts plant defences against other phytopathogens since the inoculation of *Streptomyces* spp. induces the expression of defense-related genes—at least, so it was found to do in a pea crop [70]. This ability could also explain why *Streptomyces* spp. are not limited to inhibition of bacterial species but also inhibit fungal phytopathogens in planta [71,72].

Therefore, in addition to combating the cold-associated pathogen *P. syringae*, *Streptomyces* sp. M2 likely contributes to the overall cold tolerance of *Brachypodium* and thus would be central to the cold-acclimated microbiome. *Streptomyces* spp. have a variety of adaptations for cold resistance, including the production of cold shock proteins and small solutes for cryoprotection [73,74,75]. These products may assist host survival, since a strain of *Streptomyces* was shown to alleviate the effects of cold stress in turfgrass [19] and drought stress in maize [76]. In addition, BioCyc genome-wide predictions indicate that *Streptomyces* sp. M2 produces key oxidative stress enzymes that can be secreted in *Streptomyces* spp. [77,78,79]. In addition, *Streptomyces* sp. M2 synthesizes cryoprotective soluble sugars that coincidentally increase rapidly in CA *Brachypodium* [6,80]. The synthesis of the osmoprotectant proline may also benefit host plants, as inoculation of sugarcane with *Streptomyces* increased proline content and drought tolerance [81]. *Streptomyces* spp. are also reported to increase drought tolerance in maize and aid in the accumulation of soluble sugars [76].

Another bacterial taxon, the genus *Solimonas*, increased 3.3-fold after CA, and although these species have a wide temperature optimum, they are characterized by polar lipids and fatty acids, which are known to contribute to cold tolerance [82]. In parallel findings, *Brachypodium* shows changes in metabolic pathways leading to restructuring of the plasma membrane after CA, a common vulnerability for both microbes and their hosts [6,83,84]. Already mentioned was the cold tolerance of the plant-beneficial fungus *Aspergillus.* More insight could be revealed by an investigation of the functional microbiomes of CA *Brachypodium*. However, due to low reads and sequencing depths, our results can only be considered preliminary (see Supplementary File S2 and Figures S7–S9). Nevertheless, in parallel with the CA *Brachypodium* plasma membrane proteome [6], microbial proteins involved in pathways that intersect with low temperature tolerance, such as the synthesis of soluble cryoprotectants, oxidative stress, and pathogen resistance, were detected in the microbiome in response to cold stress. Again, this underscored the inter-dependent and symbiotic character of the CA response.

### 4.4. Prospects and Conclusions

Taken together, both the changes in microbial community profiles following CA and the functional role of these plant beneficials suggest that commercial growers could see some benefit from the inoculation of mixed community strains, including *Streptomyces* sp. M2, for protection against *P. syringae* and other phytopathogens, while at the same time benefiting from other plant growth-promoting characteristics as well as enhancing cold resilience. With the presentation of this first CA *Brachypodium* microbiome, it is hoped that the insights gained will inspire treatment options to enhance cold tolerance and other intersecting stresses tailored toward specific agriculturally important grain crops [1,9,85,86].

This special issue of *Plants* asks, “What makes the life of stressed plants a little easier?” The answer for *Brachypodium* undergoing acclimation to low temperature in preparation for the coming winter is very clear. It is the strong partnership with a shifting above-ground bacterial and fungal community that works in concert with plant networks that intersect cold-, drought-, and antipathogen-signalling pathways to ensure that within only a few days host plants survive freeze events. Not only does it make the life of plants a “little easier”, we also argue that it may very well be essential for survival. Therefore, we propose that the battle against winter condition stresses is won by important inter-kingdom partnerships.

## Figures and Tables

**Table 1:** Similarity of percentage (SIMPER) analysis of microbiota contributing to the top ~25% of dissimilarity (Bray–Curtis) between non-acclimated (NA) and cold-acclimated (CA) samples (showing average relative abundance in %) in both leaf tissue and rhizosphere performed in PAST (v4.08).

Taxa	NA (%)	CA (%)	Average Dissimilarity	Contribution (%)	Cumulative (%)
Shotgun Rhizo (Overall Average Dissimilarity 7.1%)					
*Streptomyces* sp. M2	32.5	32.8	0.9	12.8	12.8
*Actinocatenispora sera*	5.7	7.3	0.8	11.2	24.0
*Actinocatenispora thailandica*	3.6	4.9	0.6	9.1	33.1
16S Rhizo (Overall Average Dissimilarity 10.7%)					
*Actinocatenispora*	8.1	11.4	1. 7	15.7	15.7
*Streptomyces*	24.2	26.6	1.4	13.4	29.2
ITS Rhizo (Overall Average Dissimilarity 19.0%)					
*Phialemonium*	21.3	13.4	4.0	21.1	21.1
*Apiotrichum*	29.9	30.8	4.0	21.1	42.2
Shotgun Leaf (Overall Average Dissimilarity 52. 6%)					
*Microcystis aeruginosa*	12.1	27.4	9.6	18.2	18.2
*Streptomyces* sp. M2	4.4	15.1	5.4	10.2	28.4
16S Leaf (Overall Average Dissimilarity 60.9%)					
*Pseudomonas*	19.7	1.0	9.4	15.4	15.4
*Rhodococcus*	15.0	0.4	7.3	12.0	27.4
ITS Leaf (Overall Average Dissimilarity 80.3%)					
*Aspergillus*	15.9	47.5	16.6	20.7	20.7
*Goidanichiella*	25.1	0.0	12.6	15.7	36.4

**Table 2:** Core microbiota taxa (species or distinct ASVs as indicated) present in 100% of samples for each sequencing and analysis method of shotgun, 16S rRNA, and ITS sequencing methodologies with an average relative abundance >5%.

Phyla	Class	Order	Family	Genus	Species
Core rhizosphere species (shotgun)					
Actinobacteria	Actinomycetia	*Streptomycetales*	*Streptomycetaceae*	*Streptomyces*	*Streptomyces* sp. M2
Actinobacteria	Actinomycetia	*Micromonosporales*	*Micromonosporaceae*	*Actinocatenispora*	*Actinocatenispora sera*
Core rhizosphere genera (16S)					
Actinobacteria	Actinomycetia	*Streptomycetales*	*Streptomycetaceae*	*Streptomyces*	
Actinobacteria	Actinomycetia	*Micromonosporales*	*Micromonosporaceae*	*Actinocatenispora*	
Proteobacteria	Gammaproteobacteria	*Xanthomonadales*	*Rhodanobacteraceae*	*Rhodanobacter*	
Core rhizosphere genera (ITS)					
Ascomycota					
Basidiomycota	Tremellomycetes	*Trichosporonales*	*Trichosporonaceae*	*Apiotrichum*	
Ascomycota	Sordariomycetes	*Sordariales*	*Cephalothecaceae*	*Phialemonium*	
Ascomycota	Saccharomycetes	*Saccharomycetales*	*Saccharomycetaceae*	*Candida*	
Core leaf species (shotgun)					
Actinobacteria	Actinomycetia	*Streptomycetales*	*Streptomycetaceae*	*Streptomyces*	*Streptomyces* sp. M2
Proteobacteria	Alphaproteobacteria	*Hyphomicrobiales*	*Rhizobiaceae*	*Liberibacter*	* ‘*Candidatus* L. a.’
Core leaf genera (16S)					
Actinobacteria	Actinomycetia	*Streptomycetales*	*Streptomycetaceae*	*Streptomyces*	
Proteobacteria	Gammaproteobacteria	*Xanthomonadales*	*Rhodanobacteraceae*	*Rhodanobacter*	
Proteobacteria	Gammaproteobacteria	*Salinisphaerales*	*Solimonadaceae*	*Solimonas*	
Core leaf genera (ITS)					
Unidentified Fungi					

* ‘*Candidatus* Liberibacter africanus’.

## Data Availability

All raw sequences were deposited in the National Centre for Biotechnology Information (NCBI) Sequence Read Archive (SRA) under BioProject ID: PRJNA782211, available at https://www.ncbi.nlm.nih.gov/bioproject/782211 (accessed on 21 November 2021).

## Supplementary Materials

The following are available online at https://www.mdpi.com/article/10.3390/plants10122824/s1. Supplementary File S1: Contains all scripts and commands used. Supplementary File S2: Contains all Supplemental Tables, Supplemental Figures, and Supplemental Text describing functional classification of shotgun data. Figure S1: Bulk soil collection from a farm field. Figure S2: Representative three-week-old *Brachypodium distachyon*. Figure S3: Image showing an example of the tightly bound root soil still attached to the plant. Figure S4: Read statistics of the shotgun sequencing processing for averages of the cold-acclimated and non-acclimated leaf and rhizosphere samples. Figure S5: Principal coordinate analysis plots comparing the taxonomic communities from amplicon sequencing blank kit controls. Figure S6: Correlation plots comparing shotgun sequencing to amplicon sequencing results under both non-acclimated and cold-acclimated conditions in the leaf and rhizosphere samples. Figure S7: Heatmaps showing the average relative abundance of the Pfam domains. Figure S8: Heatmap showing the top 50 most abundant MetaCyc pathways in rhizosphere samples. Figure S9: Heatmap showing the top 50 most abundant MetaCyc pathways in leaf samples. Table S1: DNA, final library concentration, and average library size. Table S2: Summary of quality control and preprocessing of metagenomic reads from shotgun sequencing. Table S3: Summary of the top ten average relative abundant taxa for rhizosphere samples showing average relative abundance for each non-acclimated and cold-acclimated conditions of shotgun, 16S, and ITS sequencing. Table S4: Summary of the top ten average relative abundant taxa for leaf samples showing average relative abundance for each non-acclimated and cold-acclimated conditions of shotgun, 16S, and ITS sequencing.
