# Supplementary material for: Cold Acclimation in Brachypodium Is Accompanied by Changes in Above-Ground Bacterial and Fungal Communities
Source: Plants (Basel). 2021 Dec 20;10(12):2824. doi: 10.3390/plants10122824 (PMC8704670; doi:10.3390/plants10122824)
Supplement: Supplementary file 1 [file plants-10-02824-s001.zip › File S2.pdf]

plants

MDPI

Supplemental files (Text, Figures Tables and References):

Cold acclimation in *Brachypodium* is accompanied by changes in above-ground bacterial and fungal communities

Collin L. Juurakko [1*], George C. diCenzo [1] and Virginia K. Walker [1,2]

[1] Department of Biology, Queen's University, Kingston, ON K7L 3N6, Canada; 11cj10@queensu.ca (CLJ); george.dicenzo@queensu.ca (GCD); walkervk@queensu.ca (VKW)
[2] Department of Biomedical and Molecular Sciences, and School of Environmental Studies, Queen's University, Kingston, ON K7L 3N6, Canada; walkervk@queensu.ca (VKW)
* Correspondence: 11cj10@queensu.ca; Tel.: 1 613.533.6000 Ext. 77360 (CLJ)

1. Introduction

It was hoped that additional insight into the changes in microbial communities associated with cold acclimation would be revealed by an investigation of the functional microbiomes of CA *Brachypodium*. The following includes the preliminary investigation of this effort, and is followed by supplemental photos, figures and tables in support of manuscript published in a special issue of Plants. Finally, additional figures and tables have been included to support the preliminary functional analysis (with Tables and Figures numbered according to the order of mention in the primary manuscript).

2. Materials and Methods

2.1 Functional classification

Paired end quality controlled and decontaminated reads output by Sunbeam were concatenated using the command "*cat sample_R1.fq sample_R2.fq > merged_sample.fq*" and input into HUMAnN (Version 3.0.0) [38] running MetaPhlan (Version 3.0) [**39**], Bowtie2 (Version 2.4.4) [**40**], DIAMOND (Version 2.0.11), and SAMtools (Version 1.13) [**41,42**]. Sequences were processed using the default UniRef90 database and the following parameters for MetaPhlAn: *--stat_q 0, --bt2_ps very-sensitive-local*; the following parameters for HUMAaN 3: *--nucleotide-subject-coverage-threshold 5.0, --translated-subject-coverage-threshold 5.0*; and the following parameters for and Bowtie 2: *-D 20 -R 3 -N 1 -L 20 -i S,1,0.50 --local*.

Gene families were regrouped and renamed to the uniref90_Pfam database using the *humann_regroup_table and humann_rename_table* commands. Special features including ungrouped genes and unintegrated pathways were retained by skipping normalization in favour of downstream normalization by MaAsLin2 (Version 1.6.0) [**43**]. The final renamed gene family and unnormalized pathway abundance tables were joined using the *humann_join_table* command and split into the stratified and unstratified tables using the *humann_split_table* command, the latter of which was used for differential abundance testing. Standard HUMAnN3 MetaCyc assigned metabolic pathways were used for analysis and were assigned classes based on the respective associated MetaCyc pathway superclasses. All scripts can be found in File S1.

2.2 Statistical analysis

To find differentially abundant gene families and functional pathways between conditions, MaAsLin2 was used. Raw, unnormalized abundance reads per kilobase (RPK) HUMAnN3 outputs for Pfam regrouped and renamed gene families and MetaCyc reaction pathways were input into MaAsLin2 and MaAsLin2 native total sum scaling normalization method was used for normalization. MaAsLin2 was used with default parameters

following a linear model (LM) analysis with log-transformation (LOG), total sum scaling (TSS), without standardization, and post-test correction using the Benjamini-Hochberg method. MaAsLin2 output log-fold change coefficients were converted to log2 fold-changes. MaAsLin2 outputs were parsed to remove any low abundant gene families (<0.01%) and pathways (<0.001%) from differential abundance results.

3. Results

3.1 Functional microbiome

Gene families that were identified according to the UniRef90 database and regrouped according to most gene features (~70% of all gene families in all samples) were compared to other databases (KEGG Orthogroups, Gene Ontology, Level-4 enzyme commission categories, and EggNOG). A total of 9,002 and 3,967 unstratified Pfam domains were found across all rhizosphere and leaf samples taken from both conditions, respectively. However, an average of only 11,165 and 28,494 reads were classified for gene families for NA and CA samples, respectively. Thus, low reads cautioned further analysis and interpretation of the cold-association functional leaf microbiome.

The most abundant genes in the rhizosphere samples found by pruning the top 50 gene family features (Figure S7A) were an ABC transporter (PF0005), a transport system protein (PF00528), and regulator proteins of the tetR family (PF00440). Likewise, the leaf microbiome abundant gene sequences (Figure S7B) included a Photosystem II reaction centre X protein (PsbX) (PF06596), an ABC transporter (PF0005), and a reverse transcriptase (PF00078). These top abundant gene families in the leaf and rhizosphere microbiomes likely can be attributed to housekeeping genes shared by many of the microbes. Other gene families identified in the CA leaf microbiome include stress responsive and pathogenicity related proteins such as a thioredoxin (PF00085), HSP90 (PF02518), and a pathogenicity modulator toxin/antitoxin protein (PF15919).

A total of 557 and 169 MetaCyc pathways from rhizosphere and leaf samples, respectively, were identified by HUMAnN3. The most abundant pathways in rhizosphere samples (Figure S8) were involved in the biosynthesis of purine nucleotides and amino acids suggesting active anabolism (adenosine (PWY-7220) and guanosine (PWY-7222) deoxyribonucleotide *de novo* biosynthesis II, and L-isoleucine biosynthesis I (ILEUSYN-PWY)). The most abundant pathways in leaves (Figure S9) were suggestive of anaerobic metabolism (biosynthesis of the fatty acid, gondoate (PWY-7663), 5-aminoimidazole ribonucleotide biosynthesis II (PWY-6122), and pyruvate fermentation to isobutanol (PWY-7111). Many of the other abundant MetaCyc pathways detected in the CA leaf microbiome were involved in biosynthesis, including biosynthesis of amino acids (13), nucleoside and nucleotides (11), precursor metabolites and energy (7), cell wall (4), secondary metabolites (isoprene and terpenoid (3), sugars (2), cofactor, carrier, and vitamins (2) and one each in inorganic nutrient metabolism (sulfur), carbohydrate (starch) degradation, and fatty acid and lipid biosynthesis. Differential abundance testing on gene families and pathways were performed as described using MaAsLin2. However, due to low read counts breeding cautionary results, they were omitted.

4. Discussion

In addition to typical taxonomic analyses canonical of microbiome investigations, the use of metagenomics allows for the functional characterization of the microbiome and associated shifts under conditional changes. However, low read counts require caution. In analysis of our shotgun metagenomics data, we noted an abundance of reads mapping to biosynthesis of the cryoprotectant amino acid proline from arginine. As well, additional pathways found involve the biosynthesis of arginine from ornithine and the acetyl cycle. *Brachypodium* is also known to accumulate compatible solutes including proline, so microbiome production could supplement and aid cold tolerance both in the microbes and in the plant itself [1,6,87].

Isoprene biosynthesis via the methylerythritol pathway was also found in the CA leaf microbiomes. Isoprene is the most abundant volatile compound on Earth produced mainly by plants as a thermoregulator to protect against solar heat exposure and oxidative stress and repels microbe-grazing springtails [88-93]. There is even evidence that isoprene may stabilize membranes which is an important outcome of cold acclimation and cold tolerance [94]. Bacteria have also been shown to produce isoprene with production increasing under oxidative, salt, and low temperature stress [93,95,96]. *Brachypodium* microbiome production of isoprene likely also contributes to plant cold tolerance.

The assessment of cold-associated individual gene families also revealed stress-related proteins. Similar to the CA plasma membrane proteome of *Brachypodium*, proteins involved in additional stress responses such as oxidative stress and pathogen stress were detected in the CA leaf microbiome [6]. These results demonstrate stress-pathway cross-talk induced by cold stress in the microbiome as well as the plasma membrane proteome.

This section may be divided by subheadings. It should provide a concise and precise description of the experimental results, their interpretation, as well as the experimental conclusions that can be drawn.

References (numbered according to the main text):

1. Juurakko, C.L., diCenzo, G.C. & Walker, V.K. Cold acclimation and prospects for cold-resilient crops. *Plant Stress*, **2021**, 2, 100028.
6. Juurakko, C L., Bredow, M., Nakayama, T., Imai, H., Kawamura, Y., diCenzo, G C., ... & Walker, V K. The *Brachypodium distachyon* cold-acclimated plasma membrane proteome is primed for stress resistance. *G3*, **2021**, *11*(9), jkab198.
39. Langmead, B., & Salzberg, S.L. Fast gapped-read alignment with Bowtie 2. *Nature Methods*, **2012**, *9(4*), 357-359.
40. Buchfink, B., Xie, C., & Huson, D.H. Fast and sensitive protein alignment using DIAMOND. *Nature Methods*, **2015,** *12(1*), 59-60.
41. Li, H., Handsaker, B., Wysoker, A., Fennell, T., Ruan, J., Homer, N., ... & Durbin, R. The sequence alignment/map format and SAMtools. *Bioinformatics*, **2009**, *25(16)*, 2078-2079.
42. Danecek, P., Bonfield, J.K., Liddle, J., Marshall, J., Ohan, V., Pollard, M.O., ... & Li, H. (2021). Twelve years of SAMtools and BCFtools. *Gigascience*, **2021,** *10(2*), giab008.
43. Mallick, H., Tickle, T. L., McIver, L. J., Rahnavard, G., Nguyen, L. H., Weingart, G., ... & Subramanian, A. Multivariable association in population-scale meta'omic surveys. Submission. **2020**. Available online: https://huttenhower.sph.harvard.edu/maaslin2/ Accessed Sept. 6, 2021.
87. Colton-Gagnon, K., Ali-Benali, M.A., Mayer, B. F., Dionne, R., Bertrand, A., Do Carmo, S., & Charron, J.B. Comparative analysis of the cold acclimation and freezing tolerance capacities of seven diploid *Brachypodium distachyon* accessions. *Annals of Botany*, **2014**, *113(4)*, 681-693.
88. Affek, H.P., & Yakir, D. Protection by isoprene against singlet oxygen in leaves. *Plant Physiology*, **2002**, *129(1)*, 269-277.
89. Loreto, F., Mannozzi, M., Maris, C., Nascetti, P., Ferranti, F., & Pasqualini, S. Ozone quenching properties of isoprene and its antioxidant role in leaves. *Plant Physiology*, **2001**, *126(3),* 993-1000.
90. Loreto, F., & Velikova, V. Isoprene produced by leaves protects the photosynthetic apparatus against ozone damage, quenches ozone products, and reduces lipid peroxidation of cellular membranes. *Plant Physiology*, **2001**, *127(4*), 1781-1787.
91. Sharkey, T.D., Wiberley, A.E., & Donohue, A.R. Isoprene emission from plants: why and how. *Annals of Botany***, 2008**, *101(1)*, 5-18.
92. Behnke, K., Kleist, E., Uerlings, R., Wildt, J., Rennenberg, H., & Schnitzler, J.P. RNAi-mediated suppression of isoprene biosynthesis in hybrid poplar impacts ozone tolerance. *Tree Physiology*, **2009**, *29(5)*, 725-736.
93. McGenity, T.J., Crombie, A.T., & Murrell, J.C. Microbial cycling of isoprene, the most abundantly produced biological volatile organic compound on Earth. *The ISME Journal*, **2018**, *12(4)*, 931-941.
94. Siwko, M.E., Marrink, S.J., de Vries, A.H., Kozubek, A., Uiterkamp, A.J.S., & Mark, A.E. Does isoprene protect plant membranes from thermal shock? A molecular dynamics study. *Biochimica et Biophysica Acta -Biomembranes*, **2007**, *1768*(2), 198-206.
95. Kuzma, J., Nemecek-Marshall, M., Pollock, W.H., & Fall, R. Bacteria produce the volatile hydrocarbon isoprene. Current *Microbiology*, **1995**, *30(2)*, 97-103.
96. Guenther, A.B., Jiang, X., Heald, C.L., Sakulyanontvittaya, T., Duhl, T., Emmons, L.K., & Wang, X. The Model of emissions of gases and aerosols from nature version 2.1 (MEGAN2. 1): an extended and updated framework for modeling biogenic emissions. *Geoscientific Model Development*, **2012**, *5(6)*, 1471-1492.

Table S1. DNA, final library concentration, and average library size. An asterisk (*) denotes microbial enriched DNA following the depletion of eukaryotic DNA as described.

Sample ID	DNA concentration (ng/μL)	Final library DNA concentration (ng/μL)	Average Library size (bp)
CA-leaf-1	2.3*	15.40	608
CA-leaf-2	2.4*	10.60	608
CA-leaf-3	2.5*	16.50	646
NA-leaf-1	3.5*	14.60	606
NA-leaf-2	2.6*	16.10	606
NA-leaf-3	3.5*	9.9	648
CA-rhizo-1	102.0	11.1	717
CA-rhizo-2	116.0	16.9	639
CA-rhizo-3	79.4	7.9	603
NA-rhizo-1	15.5	9.7	600
NA-rhizo-2	65.8	15.5	624
NA-rhizo-3	53.2	14.5	651

Table S2. Summary of quality control and preprocessing of metagenomic reads from shotgun sequencing.

Sample	Raw Reads (%)	Reads Surviving QC (%)	Reads Surviving Host De-contamination (%)	Reads Classified by Kraken2 (%)
CA Leaf 1	9409953 (100%)	8496666 (90.3%)	104356 (1.2%)	16126 (15.5%)
CA Leaf 2	9830288 (100%)	8967385 (91.2%)	109563 (1.2%)	11846 (10.8%)
CA Leaf 3	9137115 (100%)	8270119 (90.5%)	93053 (1.1%)	8461 (9.1%)
NA Leaf 1	10047365 (100%)	9153699 (91.1%)	89410 (1.0%)	5353 (6.0%)
NA Leaf 2	7366660 (100%)	6692997 (90.9%)	61243 (0.9%)	2429 (4.0%)
NA Leaf 3	12393210 (100%)	11269580 (90.9%)	105675 (0.9%)	4696 (4.4%)
CA Rhizo 1	12020385 (100%)	9794561 (81.5%)	9786516 (99.9%)	5404533 (55.2%)
CA Rhizo 2	10849901 (100%)	8944484 (82.4%)	8928456 (99.8%)	5113964 (57.3%)
CA Rhizo 3	11008698 (100%)	9059067 (82.3%)	9028296 (99.7%)	5082933 (56.3%)
NA Rhizo 1	11110691 (100%)	9219665 (83.0%)	9189501 (99.7%)	4500836 (49.0%)
NA Rhizo 2	10571458 (100%)	8425259 (79.7%)	8382652 (99.5%)	4719643 (56.3%)
NA Rhizo 3	13198619 (100%)	10680547 (80.9%)	10651966 (99.7%)	5904533 (55.4%)

Table S3. Summary of the top ten average relative abundant taxa, ordered from the greatest to the least, for rhizosphere samples showing average relative abundance for each non-acclimated and cold-acclimated conditions of shotgun, 16S, and ITS sequencing.

Shotgun		16S		ITS	
**Non-acclimated**	**Cold-acclimated**	**Non-acclimated**	**Cold-acclimated**	**Non-acclimated**	**Cold-acclimated**
Streptomyces sp. M2 (32.5%)	Streptomyces sp. M2 (32.8%)	Streptomyces (23.5%)	Streptomyces (29.3%)	Ascomycota (32.5%)	Apiotrichum (31.3%)
Actinocatenispora sera (5.7%)	Actinocatenispora sera (7.3%)	Rhodanobacter (7.3%)	Actinocatenispora (12.9%)	Apiotrichum (29.6%)	Ascomycota (30.2%)
Actinocatenispora thailandica (3.6%)	Actinocatenispora thailandica (4.9%)	Actinocatenispora (6.7%)	Rhodanobacter (6.9%)	Phialemonium (21.8%)	Phialemonium (13.0%)
Rhodanobacter denitrificans (1.5%)	Rhodanobacter denitrificans (1.2%)	Micropepsaceae (5.2%)	Micropepsaceae (3.7%)	Penicillium (6%)	Candida (10.4%)
Rhodanobacter sp. FDAARGOS 1247 (1.2%)	Rhodanobacter sp. FDAARGOS 1247 (1.0%)	Actinomadura (2.8%)	Chitinophagaceae (2.7%)	Candida (3.4%)	Penicillium (3.3%)
Mycobacterium colombiense (0.8%)	Mycobacterium colombiense (0.9%)	Rhizobiaceae (2.6%)	Actinomadura (2.2%)	Oidiodendron (3%)	Trichoderma (2.7%)
Mycobacterium mantenii (0.7%)	Mycobacterium mantenii (0.9%)	Chitinophagaceae (2.5%)	Rhizobiaceae (2.2%)	Trichoderma (1.1%)	Fusarium (2.4%)
Streptomyces sp. SN-593 (0.6%)	Streptomyces sp. SN-593 (0.7%)	Xanthobacteraceae (2.3%)	Xanthobacteraceae (1.9%)	Chrysosporium (0.7%)	Pseudogymnoascus (1.8%)
Rhodanobacter glycinis (0.5%)	Kribbella flavida (0.6%)	Arachidicoccus (1.7%)	Arachidicoccus (1.8%)	Fusarium (0.6%)	Chrysosporium (1.5%)
Kribbella flavida (0.5%)	Kribbella qitaiheensis (0.4%)	Rhodopirellula (0.5%)	Rhodopirellula (1.8%)	Eurotiales (0.3%)	Unidentified Fungi (0.8%)

Table S4. Summary of the top ten average relative abundant taxa, ordered from the greatest to the least, for leaf samples showing average relative abundance for each non-acclimated and cold-acclimated conditions of shotgun, 16S, and ITS sequencing.

Shotgun		16S		ITS	
**Non-acclimated**	**Cold-acclimated**	**Non-acclimated**	**Cold-acclimated**	**Non-acclimated**	**Cold-acclimated**
Microcystis aeruginosa (12.1%)	Microcystis aeruginosa (27.4%)	Pseudomonas (21.0%)	Solimonas (19.5%)	Goidanichiella (28.8%)	Aspergillus (46.1%)
Pseudomonas syringae (8.2%)	Streptomyces sp. M2 (15.1%)	Rhodococcus (14.9%)	Rhodanobacter (14.8%)	Phialemonium (17.5%)	Fusarium (17.5%)
Candidatus Liberibacter africanus (7.4%)	Candidatus Liberibacter africanus (4.3%)	Rhodanobacter (9.9%)	Streptomyces (14.3%)	Aspergillus (13.8%)	Polyporales (15.7%)
Rhodococcus qingshengii (5.0%)	Rhodanobacter glycinis (4.0%)	Streptomyces (7.9%)	Nocardioides (5.7%)	Unidentified Fungi (12.4%)	Penicillium (9.3%)
Klebsiella pneumoniae (4.9%)	Aeromonas caviae (3.3%)	Solimonas (6.2%)	Nocardioidaceae (5.1%)	Fusicolla (7.9%)	Cyberlindnera (4.4%)
Salmonella enterica (4.6%)	Microcystis viridis (3.0%)	Xanthobacter-aceae (3.0%)	Arachidicoccus (4.8%)	Saccharomyces (4.8%)	Antrodia (4.4%)
Streptomyces sp. M2 (4.4%)	Microcystis sp. MC19 (2.9%)	Nocardioides (2.8%)	Taibaiella (2.1%)	Exophiala (3.6%)	Unidentified Fungi (1.5%)
Parvularcula bermudensis (4.1%)	Salmonella enterica (2.7%)	Actinocaten-ispora (1.9%)	Methylobacte-rium-Methylo-rubrum (1.9%)	Polyporales (3.5%)	Steccherinaceae (0.6%)
Staphylococcus aureus (4.0%)	Parvularcula bermuden-sis (2.4%)	Rhizobiaceae (1.8%)	Actinocaten-ispora (1.8%)	Fusarium (3.4%)	Cephaloascus (0.4%)
Terasakiella sp. SH-1 (2.9%)	Actinocatenispora sera (2.2%)	Ralstonia (1.4%)	Ralstonia (1.6%)	Phanerochaete (2.3%)	Ascomycota (0.04%)

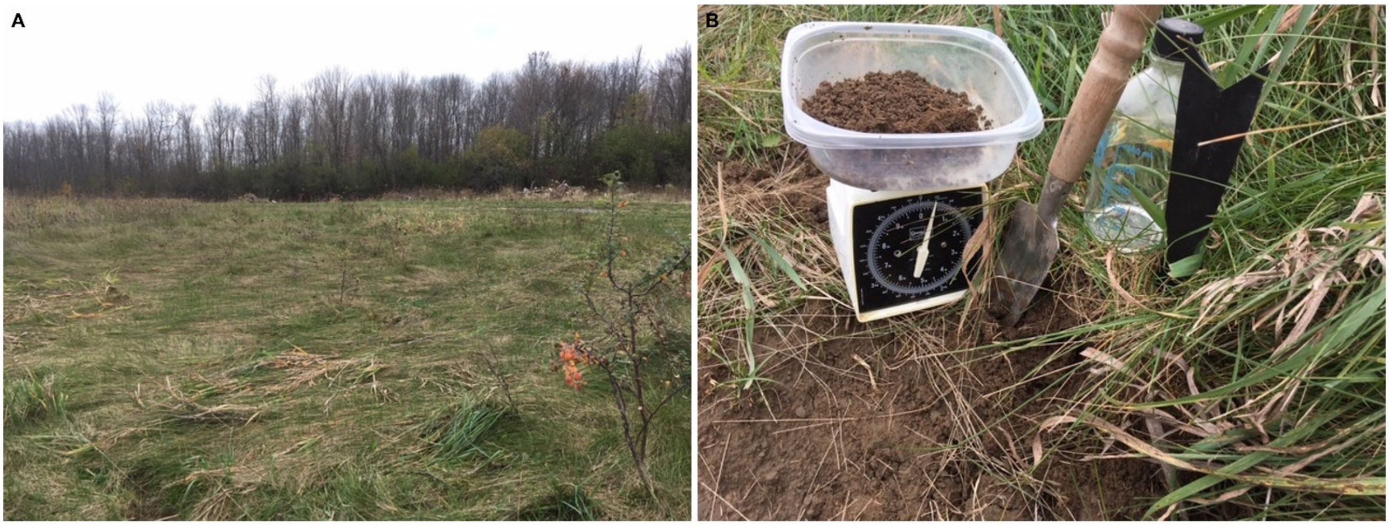

Figure S1. Bulk soil collection from a farm field north of Sydenham, Ontario, Canada (44° 24′ 26″ N, 76° 36′ 1″ W). The field **(A)** contained monocot grasses including *Dactylis*, *Bromus*, and *Phleum* that had not been fertilized or plowed in 26 years. Soil was taken using sterilized tools from the **(B)** active layer (3-7 cm deep).

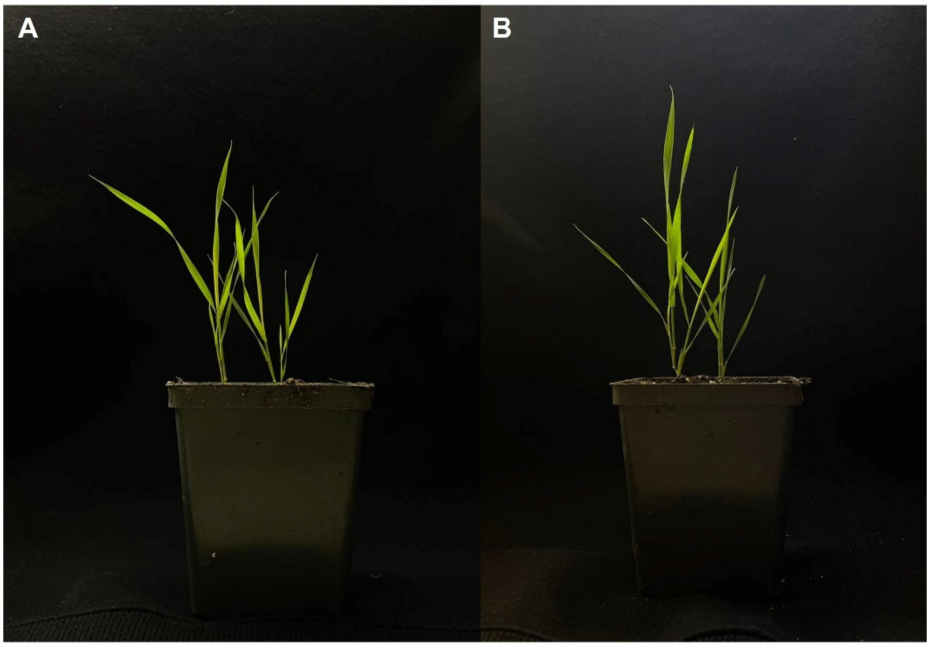

Figure S2. Representative three-week-old *Brachypodium distachyon* (inbred line ecotype Bd21) photographed at the time of use for **(A)** cold-acclimation and **(B)** non-acclimated leaf tissue and rhizosphere samples.

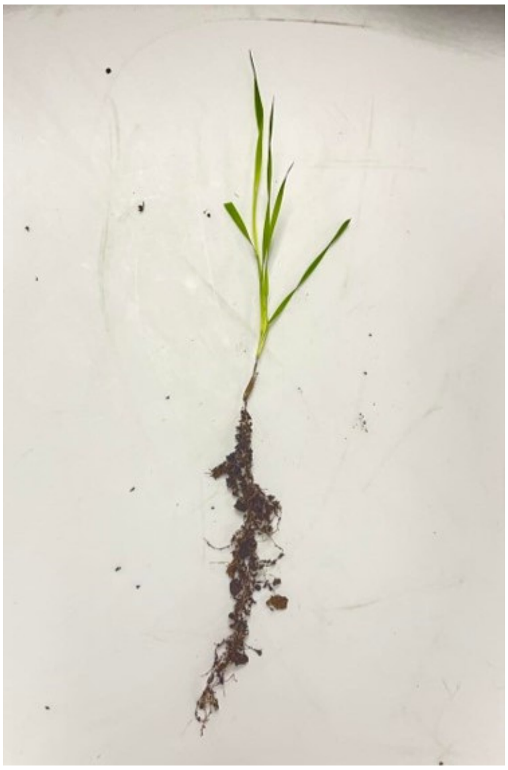

Figure S3. Image showing an example of the tightly bound root soil still attached to the plant but after shaking from the root. This soil was used for DNA extraction as described in the main text Materials and Methods, which then represented rhizosphere samples.

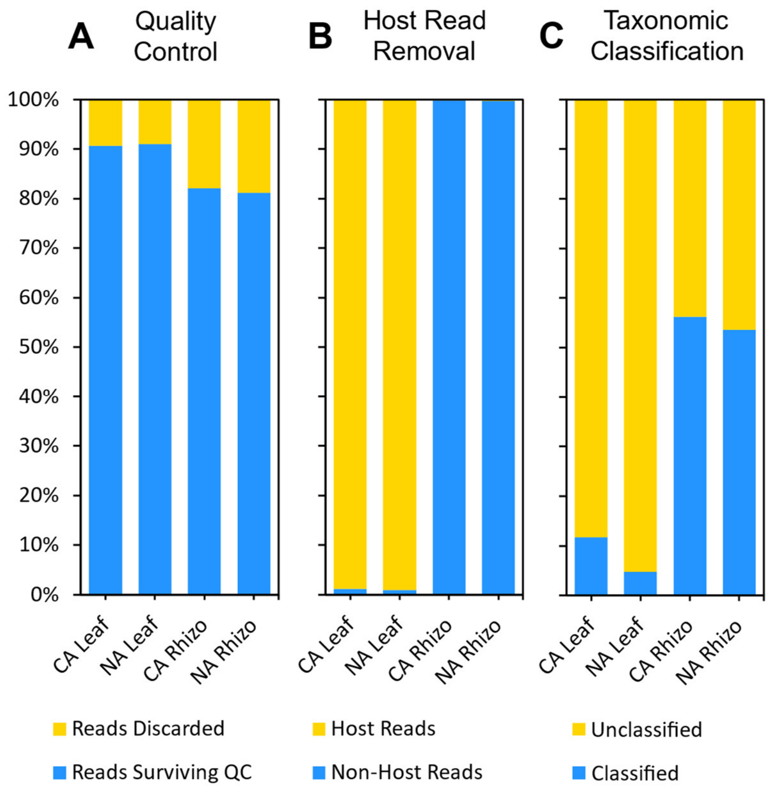

Figure S4. Read statistics of the shotgun sequencing processing for averages of the cold-acclimated and non-acclimated leaf and rhizosphere samples: **(A)** Quality control pre-processing of reads showing ratio of reads discarded to reads surviving the quality control process, **(B)** Ratio of host-contaminating reads using the *Brachypodium distachyon* genome and related grass mitochondria determined by subjecting the sequences to the BLAST algorithm (see Methods) in order to quality control reads and distinguish them from non-host reads, and **(C)** Ratio of the reads classified to the reads unclassified by the custom Kraken2 database.

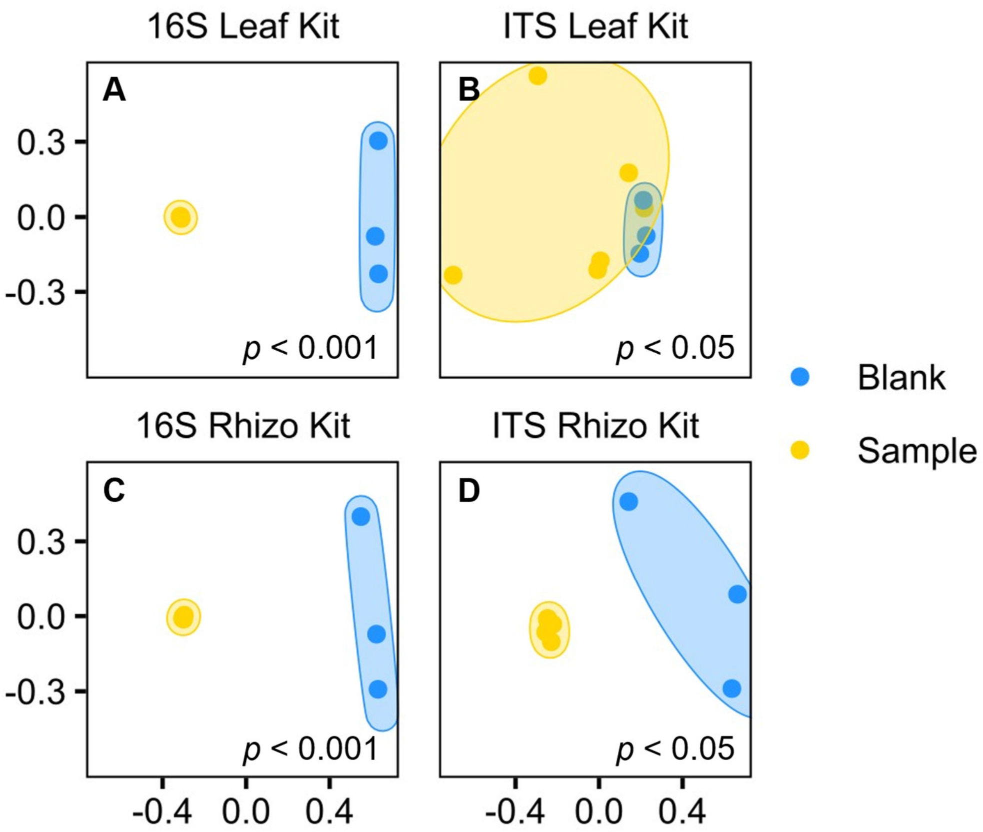

Figure S5. Principal coordinate analysis plots comparing the taxonomic communities from amplicon sequencing blank kit controls to corresponding samples extracted using the kit and subject to the same sequencing and processing pipeline: **(A)** 16S rRNA leaf taxa and blank kit using DNEasy Plant Pro Kits, **(B)** ITS leaf taxa and blank kit using DNEasy Plant Pro Kits, **(C)** 16S rRNA leaf taxa and blank kit using DNEasy PowerSoil Pro Kits, and **(D)** ITS leaf taxa and blank kit using DNEasy PowerSoil Pro Kits. Pairwise PERMANOVAs were conducted between conditions with significance noted.

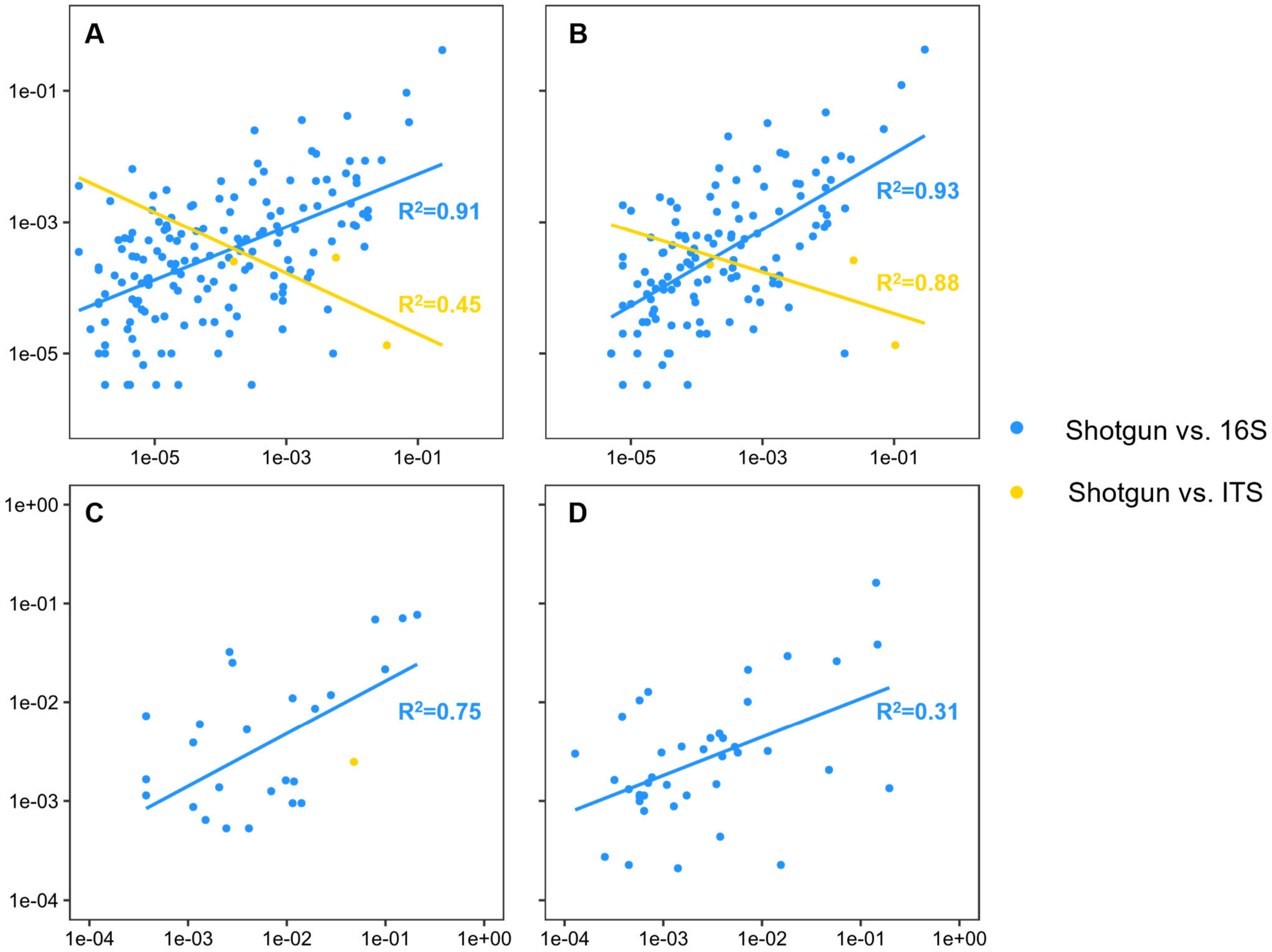

Figure S6. Correlation plots comparing shotgun sequencing to amplicon sequencing (showing shotgun *vs.* 16S rRNA and ITS in blue and yellow, respectively), results under both non-acclimated and cold-acclimated conditions in the leaf and rhizosphere samples at the genus level with linear regression lines and R^2 values shown. **(A)** Comparison of non-acclimated genera in the rhizosphere. **(B)** Comparison of the cold-acclimated genera in the rhizosphere. **(C)** Comparison of the non-acclimated genera in the leaf. **(D)** Comparison of the cold-acclimated genera in the leaf. Only genera found present in both sample sets were included.

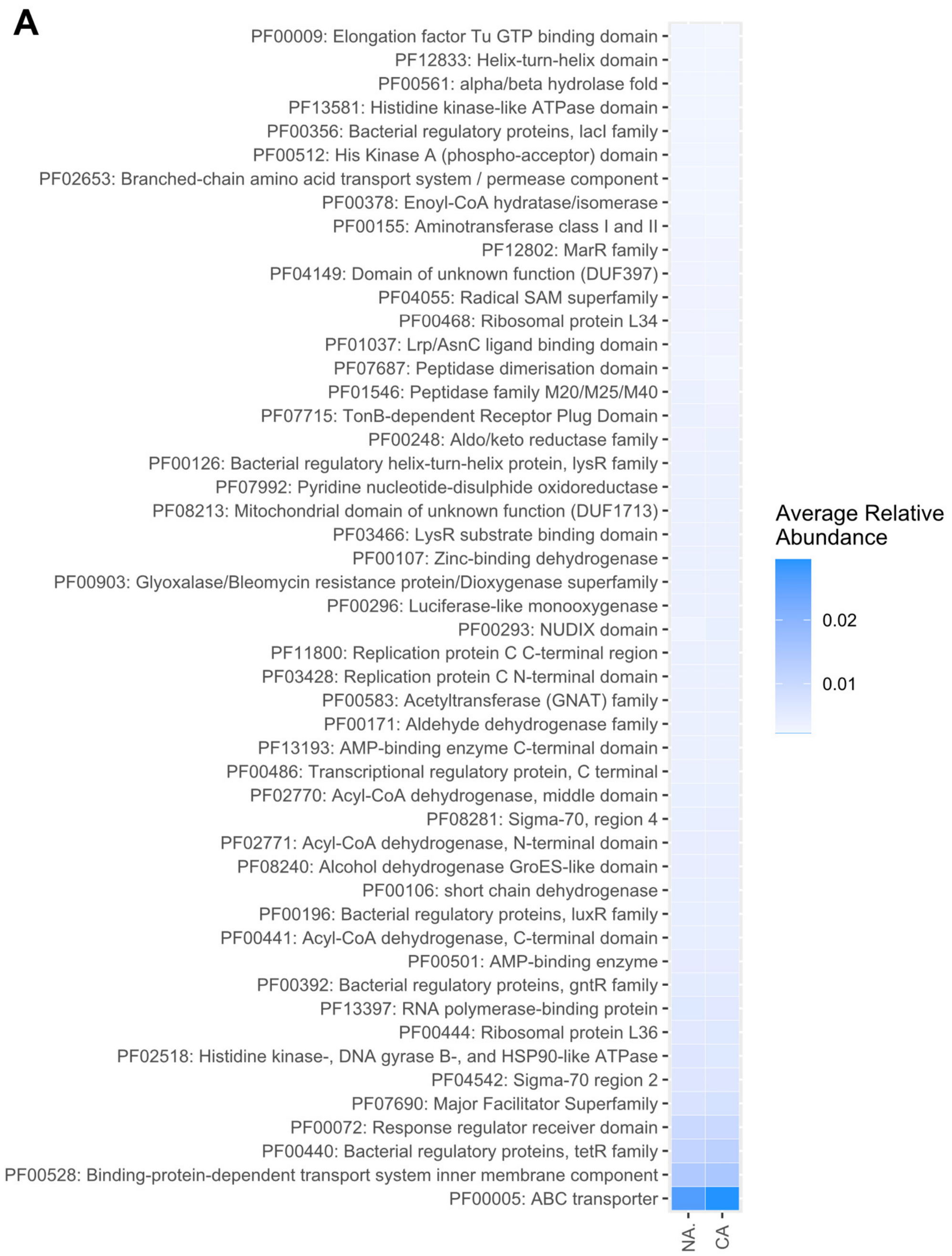
A
PF00009: Elongation factor Tu GTP binding domain
PF12833: Helix-turn-helix domain
PF00561: alpha/beta hydrolase fold
PF13581: Histidine kinase-like ATPase domain
PF00356: Bacterial regulatory proteins, lacI family
PF00512: His Kinase A (phospho-acceptor) domain
PF02653: Branched-chain amino acid transport system / permease component
PF00378: Enoyl-CoA hydratase/isomerase
PF00155: Aminotransferase class I and II
PF12802: MarR family
PF04149: Domain of unknown function (DUF397)
PF04055: Radical SAM superfamily
PF00468: Ribosomal protein L34
PF01037: Lrp/AsnC ligand binding domain
PF07687: Peptidase dimerisation domain
PF01546: Peptidase family M20/M25/M40
PF07715: TonB-dependent Receptor Plug Domain
PF00248: Aldo/keto reductase family
PF00126: Bacterial regulatory helix-turn-helix protein, lysR family
PF07992: Pyridine nucleotide-disulphide oxidoreductase
PF08213: Mitochondrial domain of unknown function (DUF1713)
PF03466: LysR substrate binding domain
PF00107: Zinc-binding dehydrogenase
PF00903: Glyoxalase/Bleomycin resistance protein/Dioxygenase superfamily
PF00296: Luciferase-like monooxygenase
PF00293: NUDIX domain
PF11800: Replication protein C C-terminal region
PF03428: Replication protein C N-terminal domain
PF00583: Acetyltransferase (GNAT) family
PF00171: Aldehyde dehydrogenase family
PF13193: AMP-binding enzyme C-terminal domain
PF00486: Transcriptional regulatory protein, C terminal
PF02770: Acyl-CoA dehydrogenase, middle domain
PF08281: Sigma-70, region 4
PF02771: Acyl-CoA dehydrogenase, N-terminal domain
PF08240: Alcohol dehydrogenase GroES-like domain
PF00106: short chain dehydrogenase
PF00196: Bacterial regulatory proteins, luxR family
PF00441: Acyl-CoA dehydrogenase, C-terminal domain
PF00501: AMP-binding enzyme
PF00392: Bacterial regulatory proteins, gntR family
PF13397: RNA polymerase-binding protein
PF00444: Ribosomal protein L36
PF02518: Histidine kinase-, DNA gyrase B-, and HSP90-like ATPase
PF04542: Sigma-70 region 2
PF07690: Major Facilitator Superfamily
PF00072: Response regulator receiver domain
PF00440: Bacterial regulatory proteins, tetR family
PF00528: Binding-protein-dependent transport system inner membrane component
PF00005: ABC transporter
NA.
CA
Average Relative Abundance
0.02
0.01

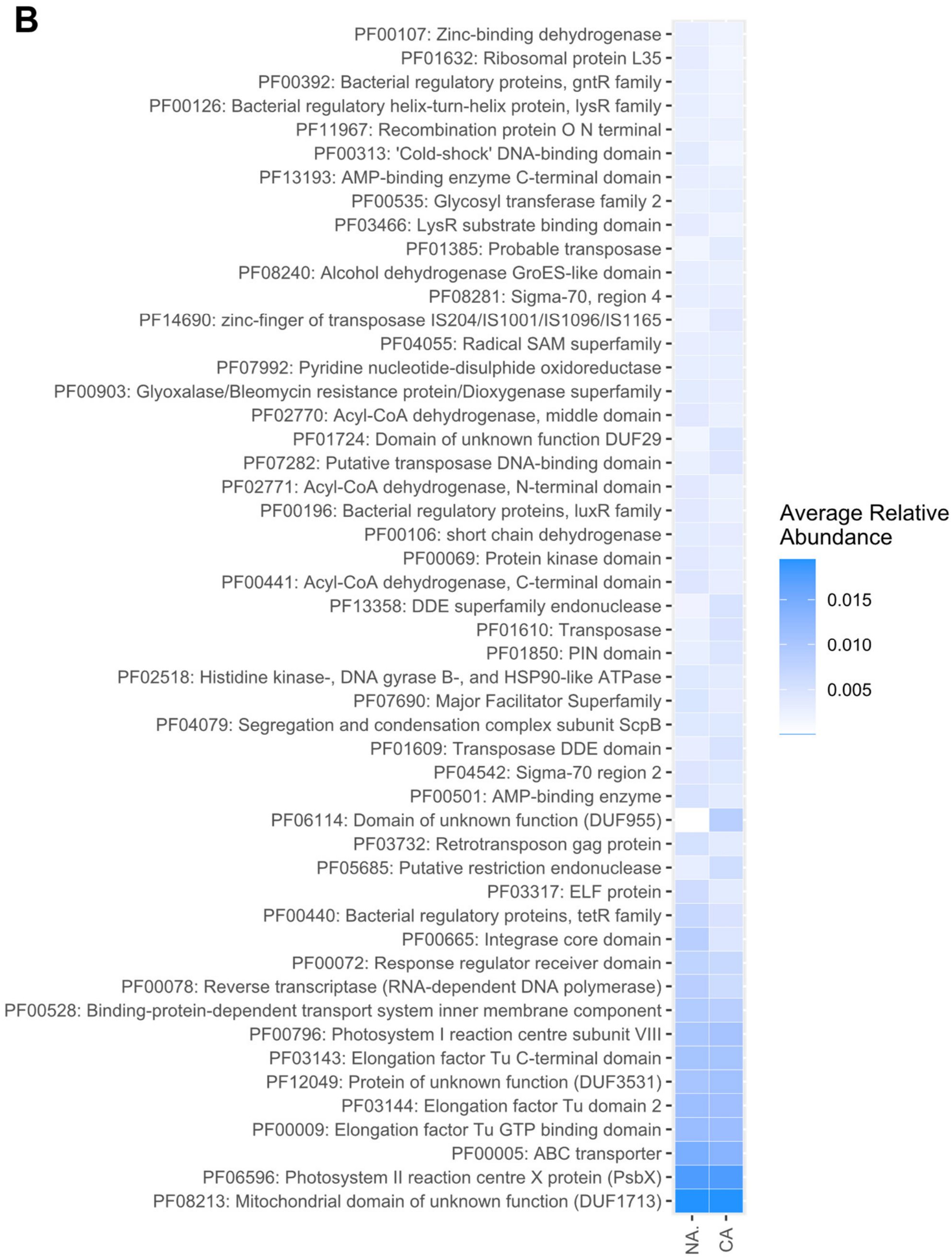

Figure S7. Heatmaps made using ggplot2 showing the average relative abundance of the top 50 most abundant Pfam domains in cold-acclimated and non-acclimated **(A)** rhizosphere samples and **(B)** leaf samples, determined using the *prune_taxa* function from the phyloseq R package.

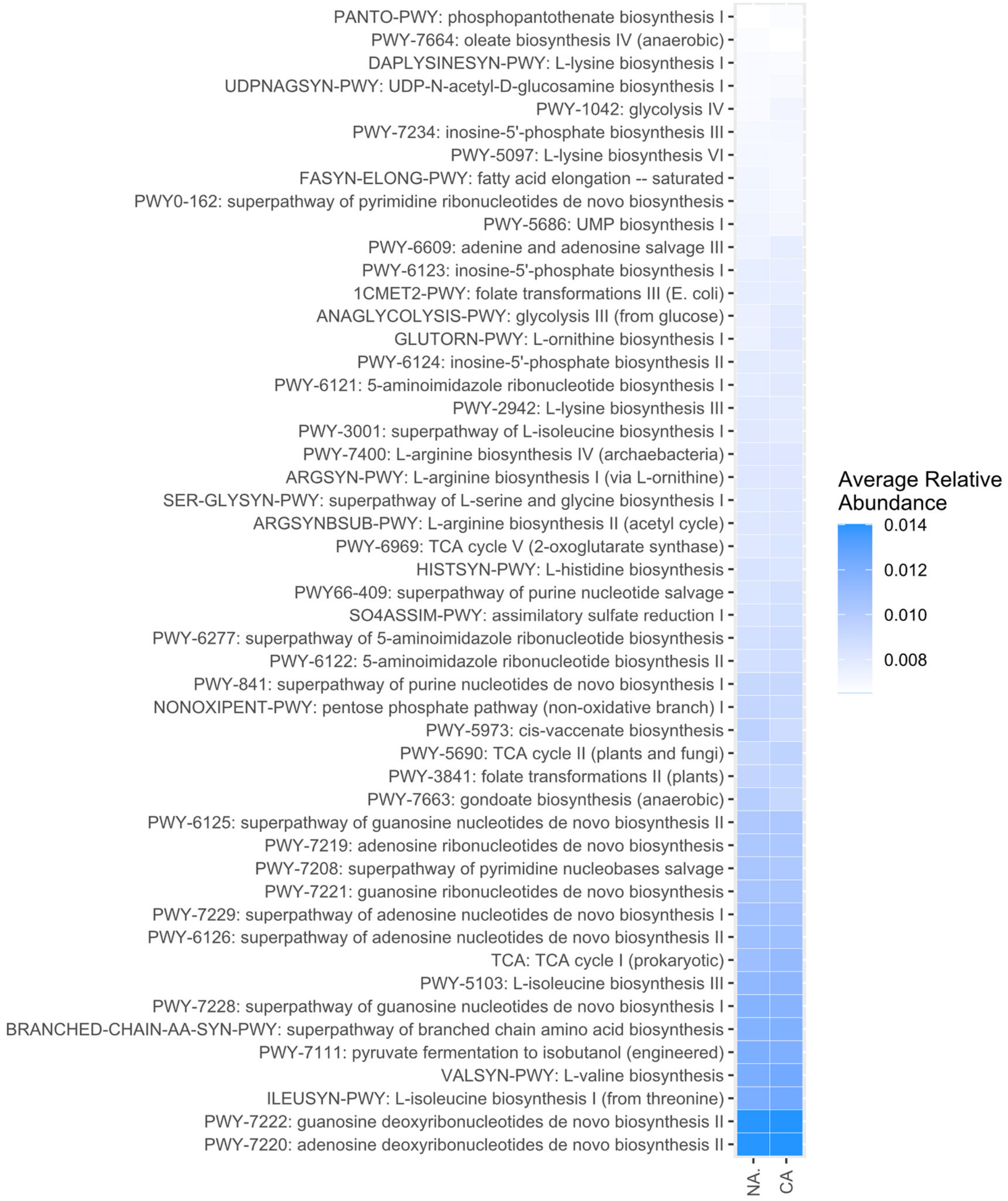

Figure S8. Heatmap made using ggplot2 showing the top 50 most abundant MetaCyc pathways in cold-acclimated and non-acclimated rhizosphere samples determined using the *prune_taxa* function from the phyloseq R package.

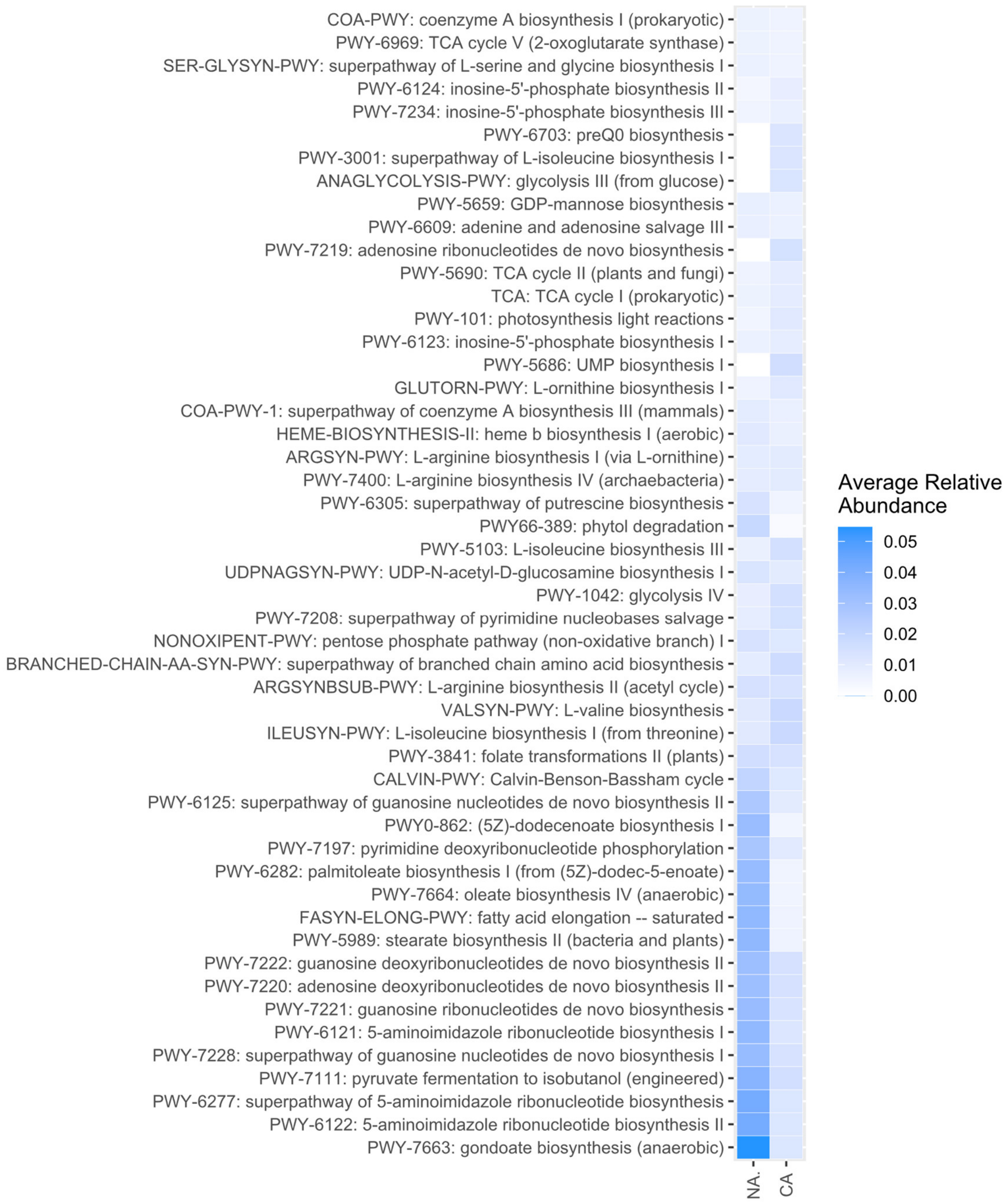

Figure S9. Heatmap made using ggplot2 showing the top 50 most abundant MetaCyc pathways in cold-acclimated and non-acclimated leaf samples determined using the *prune_taxa* function from the phyloseq R package.
